# Supplementary material for: Deep-learning models reveal how context and listener attention shape electrophysiological correlates of speech-to-language transformation
Source: PLoS Comput Biol. 2024 Nov 11;20(11):e1012537. doi: 10.1371/journal.pcbi.1012537 (PMC11581396; doi:10.1371/journal.pcbi.1012537)
Supplement: S1 Text — (PDF) [file pcbi.1012537.s001.pdf]

# Supplementary Information for:

## Deep-Learning Models Reveal How Context and Listener Attention Shape Electrophysiological Correlates of Speech-to-Language Transformation

**Andrew J. Anderson, Chris Davis, Edmund C. Lalor.**

# CONTENTS

**Fig A (Fig 1 Companion).** Illustration of the self-attention computation in a generic Transformer Encoder. **2**

**Fig B (Fig 1 Companion).** Comprehensive illustration of attention weights computed for a 3s speech segment. **3**

**Fig C (Fig 1 Companion).** Comprehensive illustration of attention weights computed for a 10s speech segment. **4**

**Fig D (Fig 1 Companion).** Exploration of how EEG prediction accuracy varies with the degree of data reduction applied to Whisper Layer 6. **5**

**Fig E (Fig 2 Companion).** Individual prediction accuracies derived with Env&Dv, MFCC and GPT-2 Lexical Surprisal. **6**

**Fig F (Fig 2 Companion).** Whisper L6 encodes almost all information in earlier layers that is valuable for modeling EEG. **7**

**Fig G (Fig 2 Companion).** Whisper captures almost all information that is useful for predicting the current EEG data in a phoneme articulation model. **8**

**Fig H (Fig 3 Companion).** EEG correlates of selectively attended and unattended speech in two concurrent speaker (audiobook) “cocktail-party” conditions. **9**

**Fig I (Fig 3 Companion).** EEG correlates of selectively attended and unattended speech in two concurrent speaker (audiobook) “cocktail-party” conditions (scalp maps). **10**

**Table A (Fig 3 Companion)** Linear Mixed Effects analysis of the effects of Selective Attention on Layer-wise EEG Scalp Average Prediction Accuracies corresponding to **Fig 3 Left**. **11**

**Fig J (Fig 4 Companion).** Exploration of how accurately representations at different layers of a language model (GPT-2-medium) predict natural speech EEG data. **12**

**Fig K (Fig 4 Companion).** Posterior scalp electrodes appear to be more sensitive to lengthier Whisper contexts. **13**

**Fig L (Fig 4 Companion).** To explore how the relative timing of EEG responses predicted by Whisper compared to the speech envelope and language model, we ran a battery of “single time lag” regression analyses. **14**

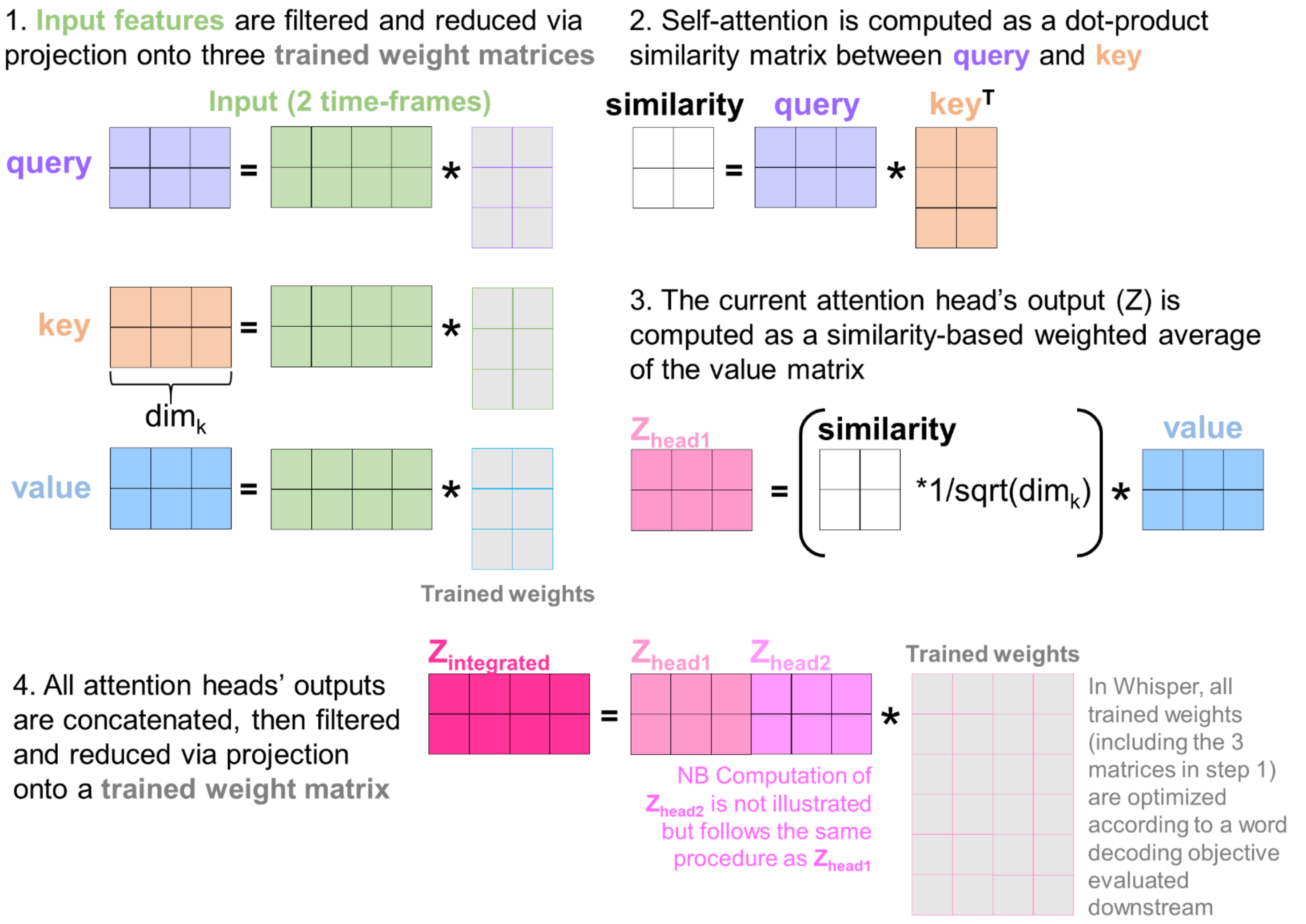


**Fig A (Fig 1 Companion).** Illustration of the self-attention computation in a generic Transformer Encoder. The illustration borrows heavily from https://jalammar.github.io/illustrated-transformer/ and uses the same nomenclature and color-coding. To simplify visualization, computation in a single layer's attention head is illustrated and layer inputs consist of only two time-frames containing four features. In practice the Whisper-base encoder has six layers, and eight separate attention heads per layer. Nonetheless, the computational procedure is the same for each layer and attention head. The initial transformer input (green) corresponds to the 80 channel Log-Mel Spectrogram, passed through a convolutional neural network, with positional codes then being added onto each time-frame to specify their relative order in the sequence. The input of successive layers is the output of the previous layer. At stage 1, information is extracted from the input, by projecting the input onto three separate trained weight matrices by matrix multiplication. The resulting representations are referred to as the query, key and value. The information extracted in the query and key matrices is critical for estimating the contextual relationships between different time-frames. At stage 2, contextual relations are estimated by multiplying the query with the transpose of the key. The resultant dot-product "similarity" matrix is populated by values that are high if query and key vectors resemble each other. The similarity matrix diagonal is liable to contain high values corresponding to the self-similarity between query and key vectors for the same time-frame. The attention head output Z is derived at stage 3. Z reflects the combination of the current time-frame with contextually-related time-frames – computed as a similarity-based weighted average of value vectors for all time frames. At stage 4, the outputs (Z) from all attention heads are concatenated, filtered and reduced through projection onto a trained weight matrix. The output $Z_{integrated}$ is fed forward for subsequent processing before forming the layer output (see https://jalammar.github.io/illustrated-transformer/ for further details).

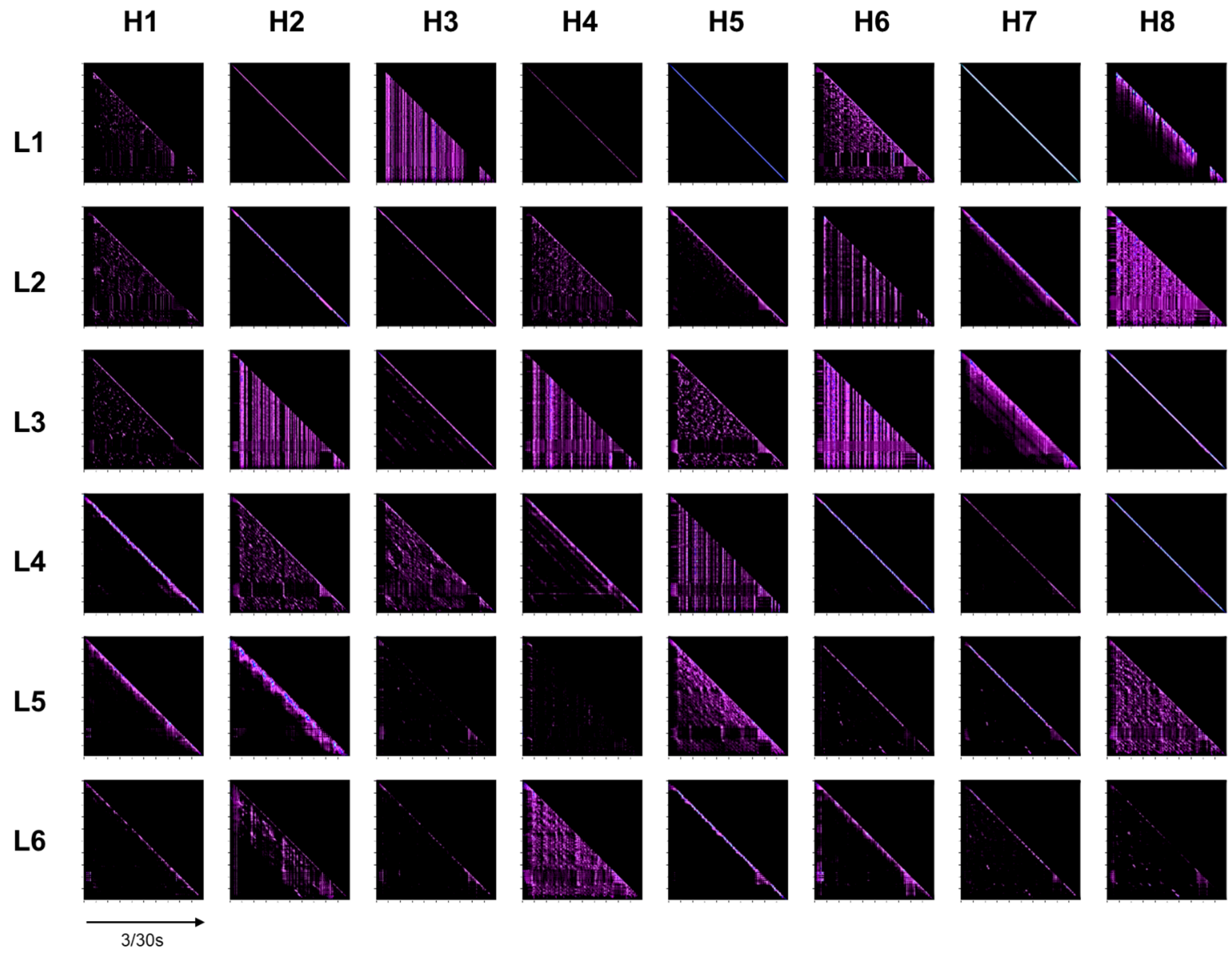


**Fig B (Fig 1 Companion).** Comprehensive illustration of attention weights computed at each layer (L) and attention head (H), for the same 3s speech segment illustrated in **Fig 1**. All attention weights are positive, or zero (black).

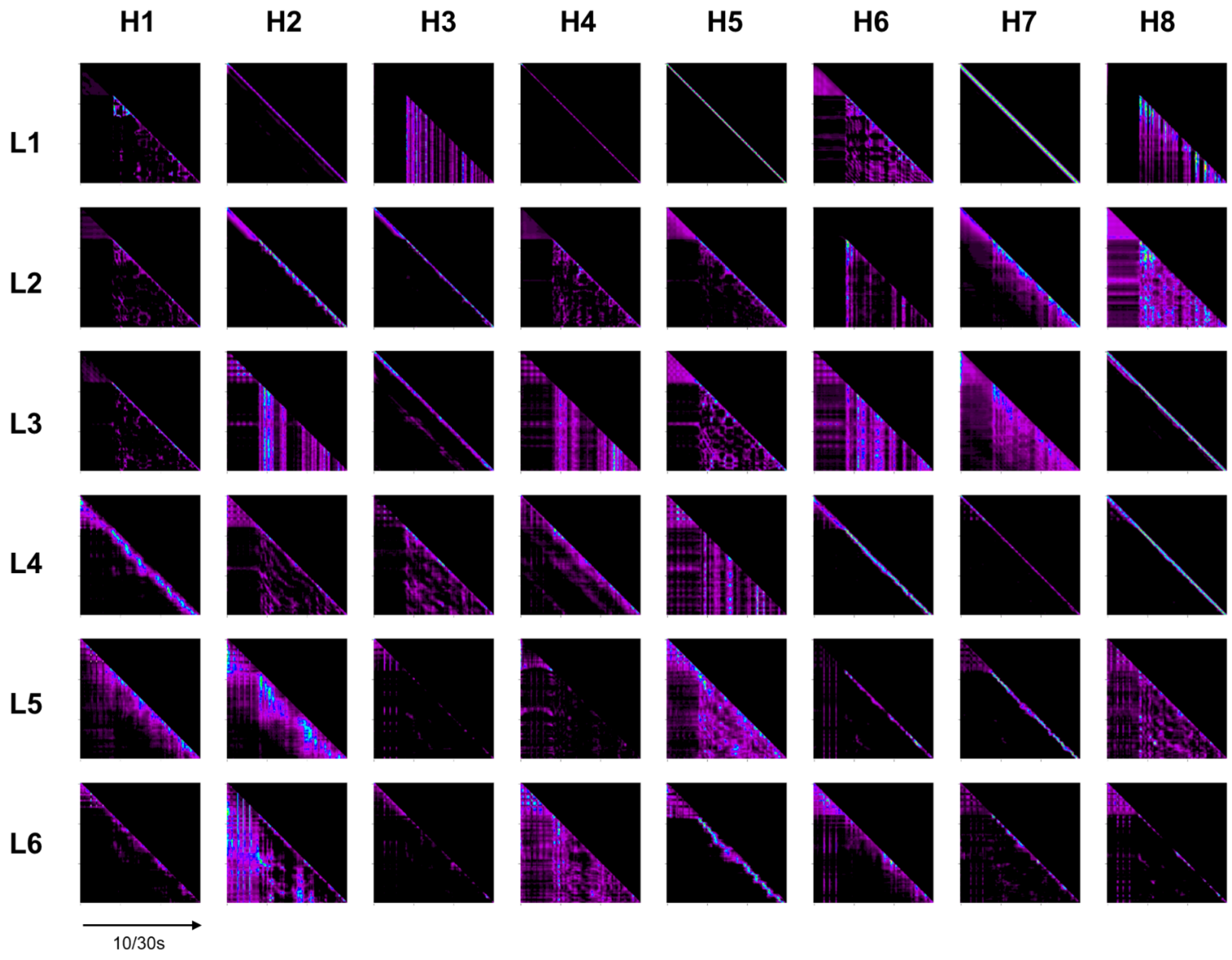


**Fig C (Fig 1 Companion).** Comprehensive illustration of attention weights computed at each layer (L) and attention head (H), for a 10s speech segment, continuing on 7s after the 3s segment illustrated in Fig 1, and Fig B in S1_Text. All attention weights are positive, or zero (black).

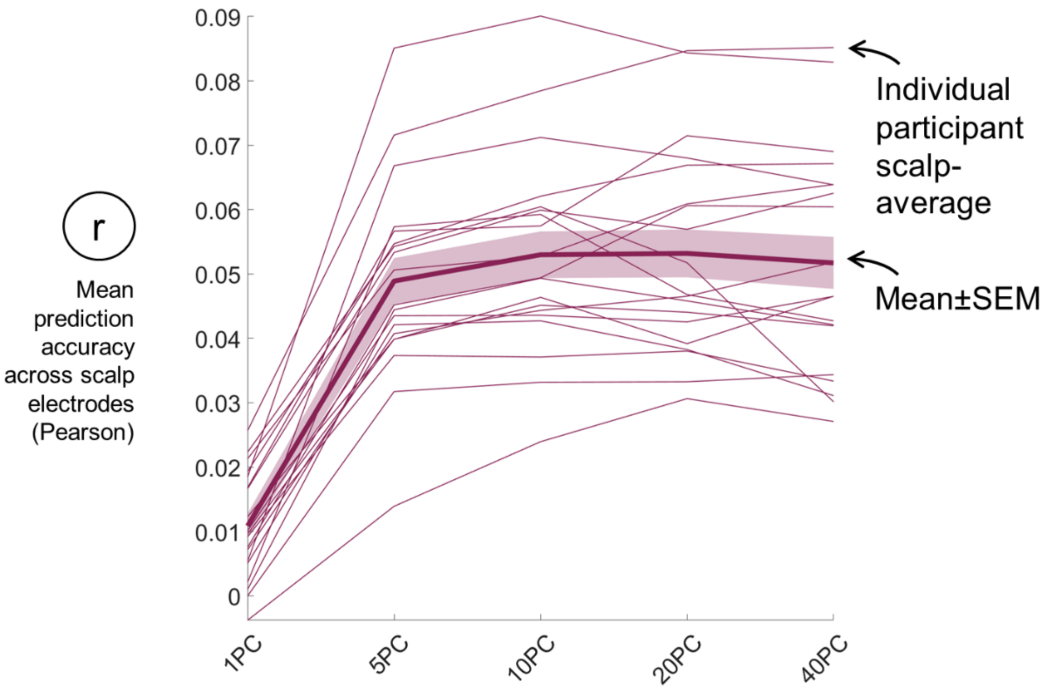


**Fig D (Fig 1 Companion).** Exploration of how EEG prediction accuracy varies with the degree of data reduction applied to Whisper Layer 6.

All Whisper-based analyses in the main article were performed following data reduction, as was achieved by projecting each Whisper layer onto a set of 10 principal component axes, that had been precomputed for each layer on Whisper representations derived from separate audiobook data. The selection of 10 components was our first choice, but arbitrary. To verify that the 10-component reduction was appropriate, the EEG data in **Fig 2** was predicted after Whisper L6 had been reduced to [1 5 10 20 and 40] components. Visual inspection of scalp-average prediction accuracies (above) suggests that accurate EEG predictions could even be obtained with 5 components, and although the most accurate predictions were derived from 40PC, the performance boost above 10PC was not pronounced.

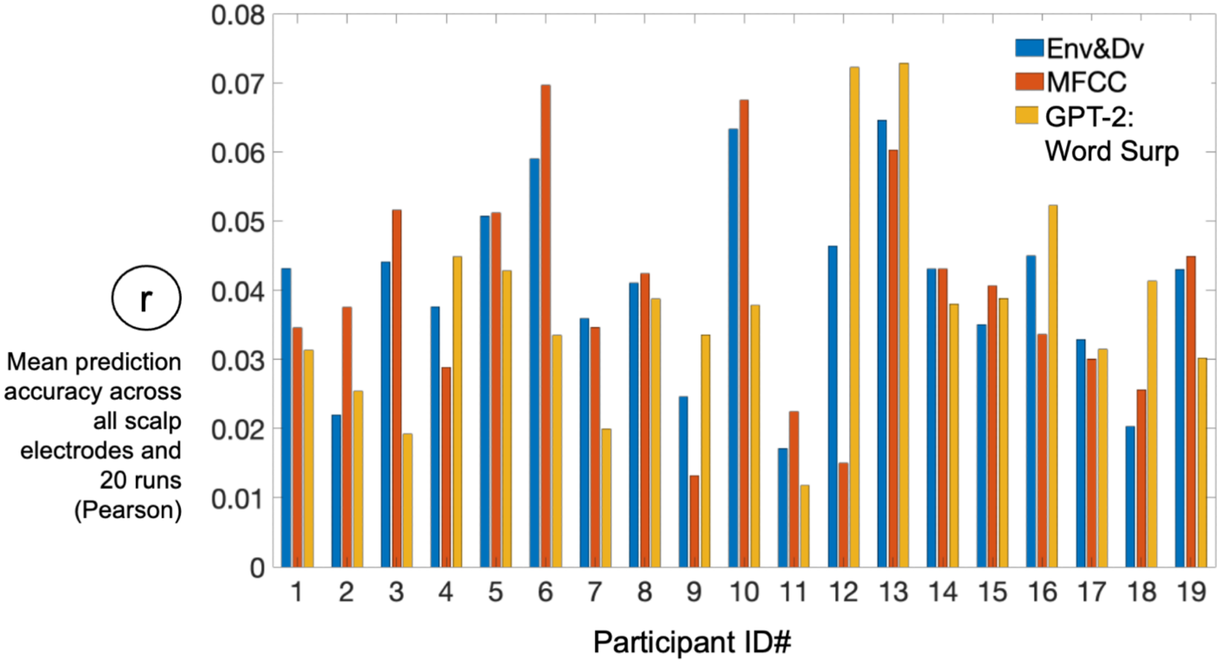


**Fig E (Fig 2 Companion).** Individual prediction accuracies derived with Env&Dv, MFCC and GPT-2 Lexical Surprisal, to complement Fig 2, where Mean±SEM only are represented in green horizontal lines. The Mean±SEM prediction accuracies displayed in Fig 2, were: Env&Dv: 0.041±0.003, MFCC: 0.039±0.004, GPT-2 Word Surprisal: 0.038±0.004.

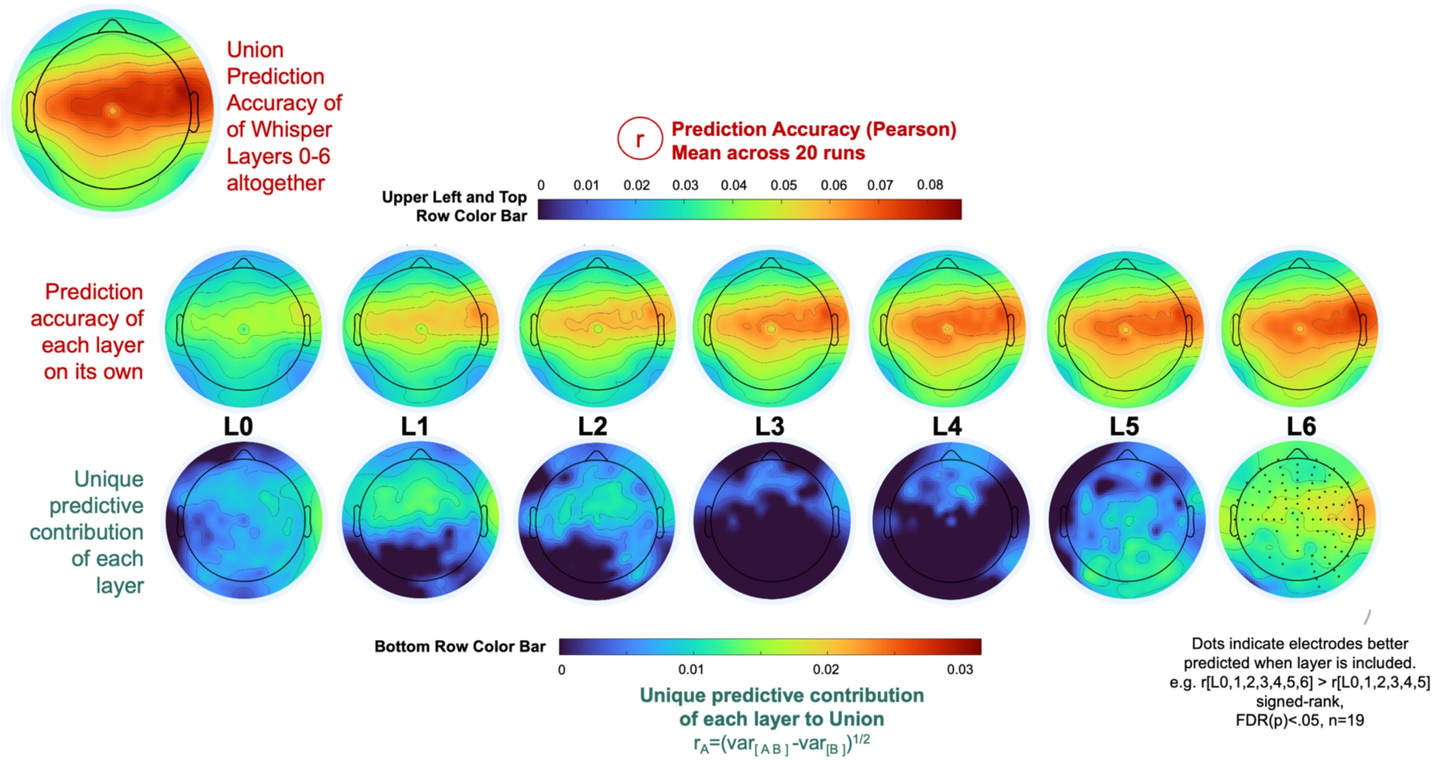


**Fig F (Fig 2 Companion).** Whisper L6 encodes almost all information in earlier layers that is valuable for modeling EEG.

**Top left:** “Union Prediction Accuracy of Whisper Layers 0-6 altogether”. Scalp map of electrode-wise EEG prediction accuracies derived from the Union of Whisper Layers L0-6 concatenated. Mean±SEM Scalp-average prediction accuracy was 0.056+/-0.004.

**Middle Row:** “Prediction accuracy of each layer on its own”. Scalp maps in the top row display electrode-wise prediction accuracies derived from Whisper Layers in isolation.

**Bottom Row:** “Unique predictive contribution of each layer”. Scalp maps in the bottom row display the unique contribution each Whisper Layer made to EEG prediction, as evaluated with predicted variance partitioning. Whisper L6 was the only layer to independently contribute to prediction. L6 Mean±SEM Scalp-average prediction accuracy was 0.053+/-0.004. This was not significantly different from scalp-average prediction accuracy derived from the Union of L0-L6 ($z=1.57$, $p=0.12$, $n=19$, Signed-Rank test).

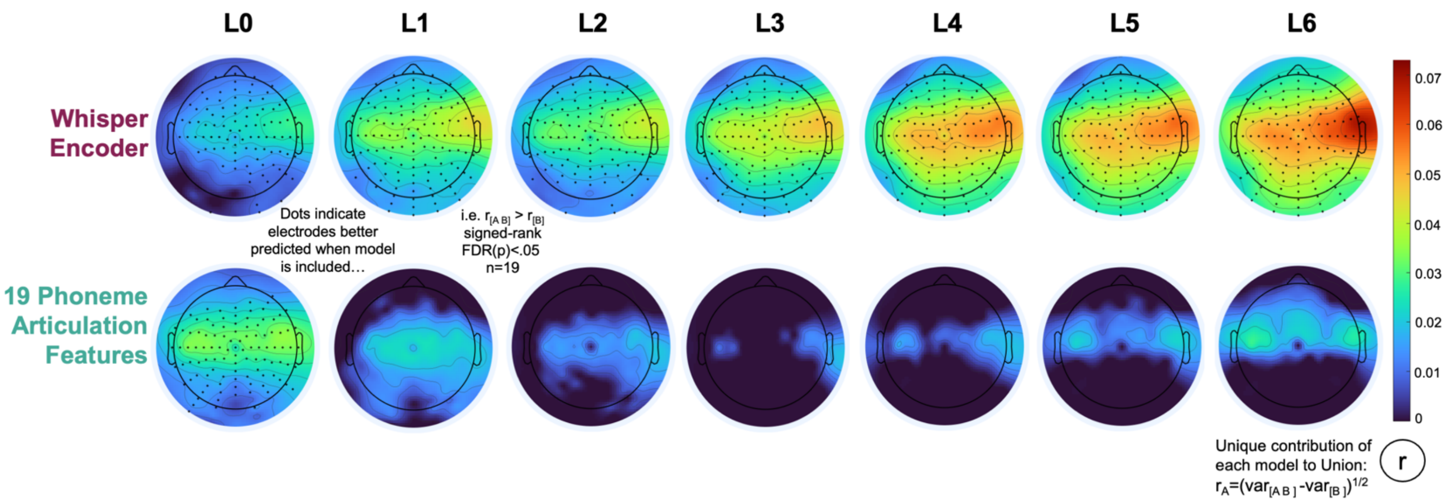


**Fig G (Fig 2 Companion).** Whisper captures almost all information that is useful for predicting the current EEG data in a phoneme articulation model. The phoneme articulation model contains 19 binary articulatory features used in [3]. EEG data was resampled at 128Hz to match the articulatory modeling set up of [3], and the 32Hz Whisper models used elsewhere in this article were up sampled to 128Hz. Time-lags used in fitting temporal response functions to both the phoneme articulation model and Whisper were 0-250ms to match the 2015 analysis. With this set up, predicted variance partitioning analyses found that Whisper layers 1 to 6 could each account for the EEG predictions made by the articulation model.

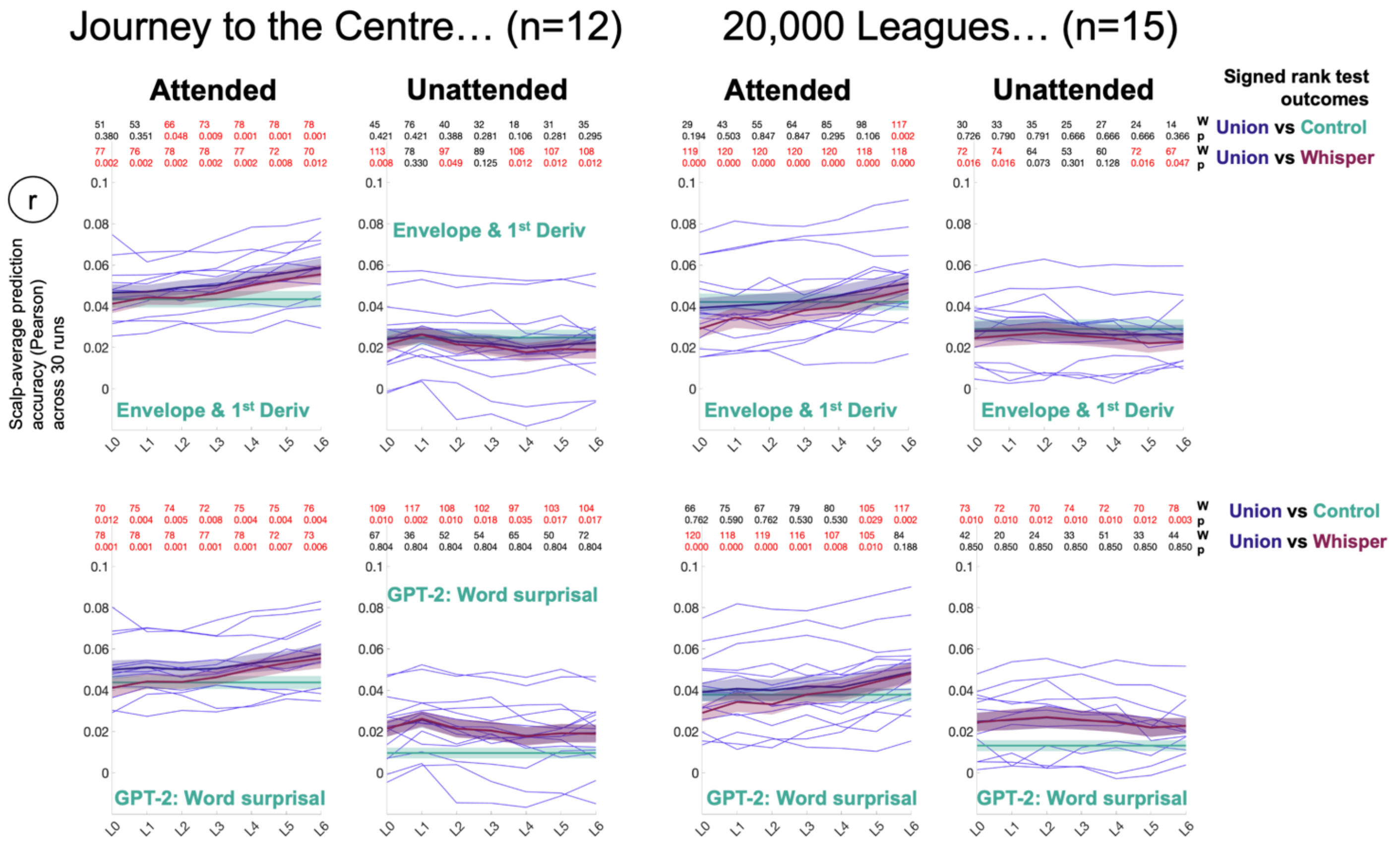


**Fig H (Fig 3 Companion).** EEG correlates of selectively attended and unattended speech in two concurrent speaker (audiobook) "cocktail-party" conditions, splitting up **Fig 3 Left** by story (Journey to the Centre of the Earth and 20,000 Leagues under the Sea).

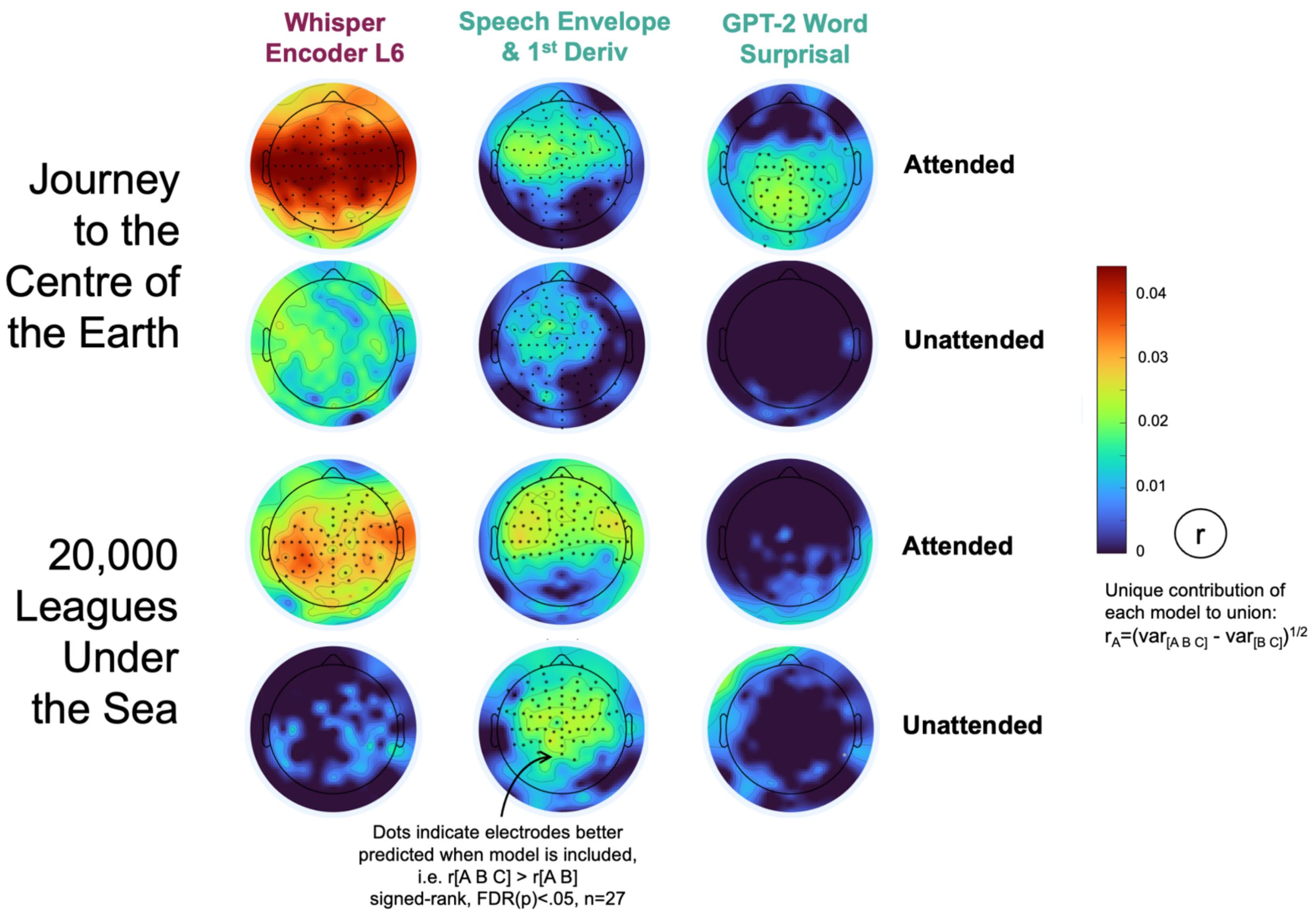


**Fig I (Fig 3 Companion).** EEG correlates of selectively attended and unattended speech in two concurrent speaker (audiobook) “cocktail-party” conditions, splitting up **Fig 3 Right** by story (Journey to the Centre of the Earth and 20,000 Leagues under the Sea).

**Table A (Fig 3 Companion)** Linear Mixed Effects analysis of the effects of Selective Attention on Layer-wise EEG Scalp Average Prediction Accuracies corresponding to **Fig 3 Left**. Model Fixed effects corresponded to Attention (nominal: Attended vs Unattended), the story heard (nominal: "Journey to…" or "20,000 Leagues…"), Whisper Layer (0 to 6), with interaction terms: Attention:Story and Attention:Layer and Attention:Story:Layer, and random effect of subject (nominal 1 to 27). The formula for the mixed model was:

Accuracy ~ 1 + Attention + Story + Layer + Attention:Story + Attention:Layer + Attention:Story:Layer + (1 | SubjectID).

Outcomes are illustrated in the Table below. Most importantly the analysis revealed a significant interaction (p=4.5e5) between selective attention and Whisper layers (deep Whisper layers accurately predicted EEG, only if the modelled speech stream was attended). This interaction is visible in **Fig 3** as the positive trend between layer depth and prediction accuracy for attended speech, comparative to the weaker negative trend when speech is unattended. Otherwise, attention was the only significant main effect (p=1.84e-45: Prediction accuracies for attended speech were greater than unattended accuracies). Finally, the interaction between attention and story was significant (p=0.002). This was because for Journey to the Centre of the Earth, the boost in prediction accuracy from unattended to attended tended to be greater than corresponding values for 20,000 Leagues Under the Sea (Journey: Mean±SEM 0.023±0.004, n=12, 20,000: Mean±SEM 0.017±0.005, n=15, when scalp-average accuracies were averaged across all layers).

| | Estimate | SEM | t | p |
|---|---|---|---|---|
| **Intercept** | -0.39705 | 0.20002 | -1.985 | 0.047883 |
| **Attention (Nominal)** | 1.1747 | 0.072004 | 16.314 | 1.8346e-45 |
| **Story (Nominal)** | -0.19423 | 0.26836 | -0.72378 | 0.46966 |
| **Layer (0 to 6)** | -0.055444 | 0.050982 | -1.0875 | 0.27752 |
| **Attention : Story** | -0.29661 | 0.096604 | -3.0704 | 0.0022958 |
| **Attention : Layer** | 0.29762 | 0.0721 | 4.128 | 4.5263e-05 |
| **Story : Layer** | -0.036779 | 0.0684 | -0.5377 | 0.59111 |
| **Attention : Story : Layer** | 0.096022 | 0.096732 | 0.99266 | 0.32153 |

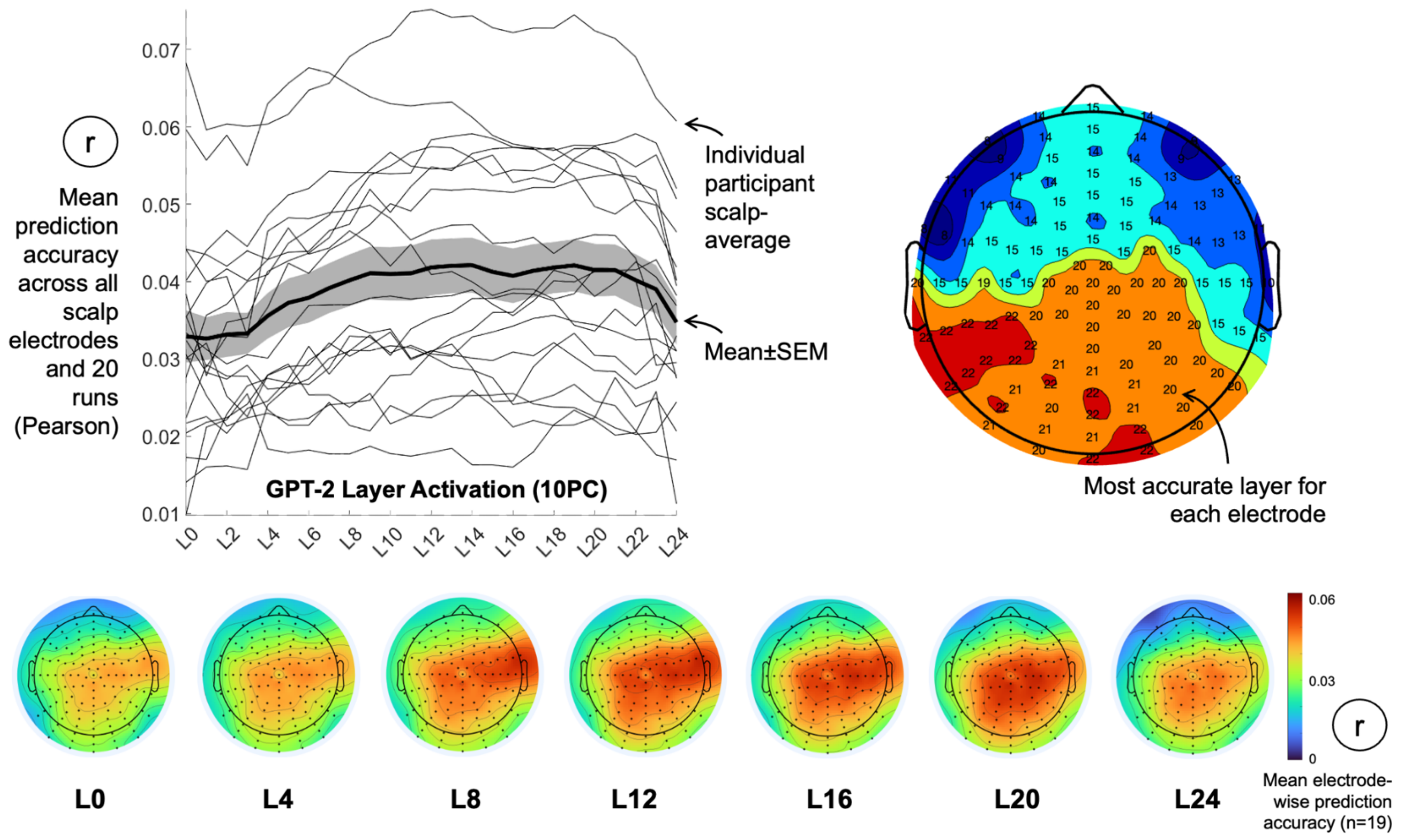


**Fig J (Fig 4 Companion).** Exploration of how accurately representations at different layers of a language model (GPT-2-medium) predict natural speech EEG data

**Top left:** Reanalysis of the audiobook EEG data from **Fig 2** found that GPT-2 scalp-average EEG prediction accuracies were visibly greater for inner layers, mirroring independent analyses of natural speech fMRI data. **Bottom:** Electrode-wise prediction accuracies derived from successive GPT-2 layers. **Top right:** Scalp color-codes indicate the GPT-2 layer that most accurately predicted each layer. The most accurate layer was determined by (1) Computing the mean prediction accuracy across participants for each layer and electrode. (2) Identifying the layer yielding the maximum mean prediction accuracy at each electrode.

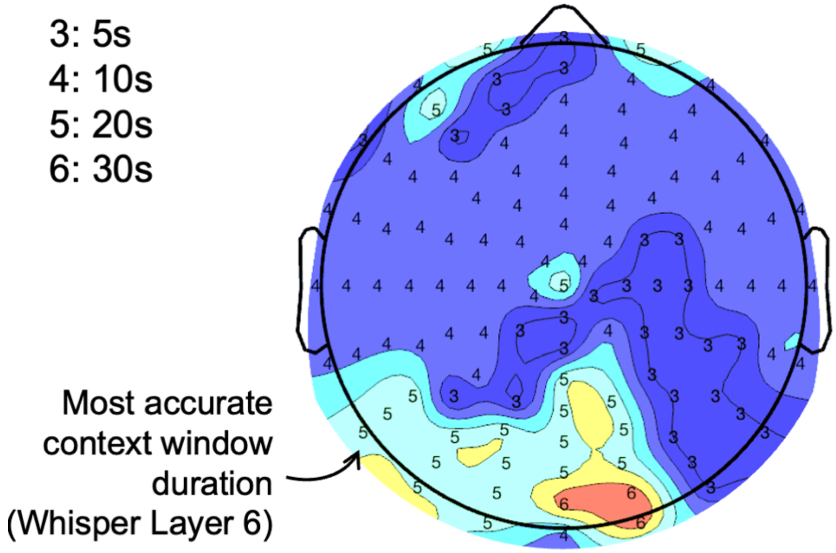


**Fig K (Fig 4 Companion).** Posterior scalp electrodes appear to be more sensitive to lengthier Whisper contexts. The scalp map displays the most accurate context window length when predicting individual electrodes with Whisper L6. The most accurate context window length was determined by taking the mean prediction accuracy across participants for each electrode, and then finding the context length with the maximum accuracy. All electrodes were preferentially predicted by 5s or more speech context.

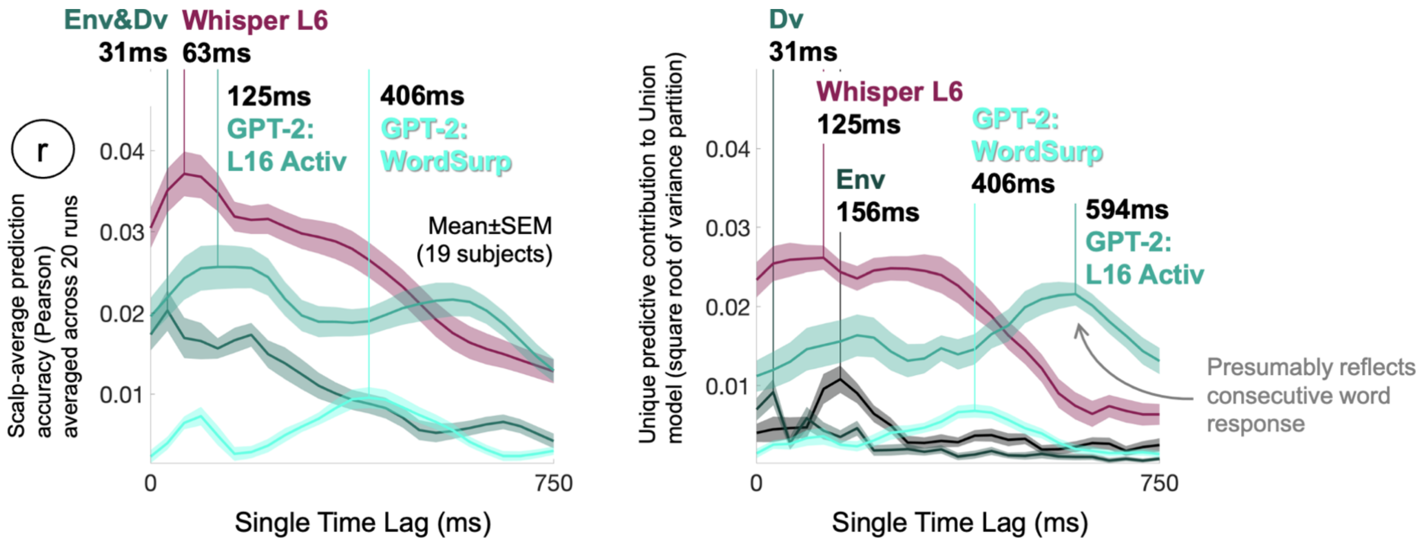


**Fig L (Fig 4 Companion).** To explore how the relative timing of EEG responses predicted by Whisper compared to the speech envelope and language model, we ran a battery of "single time lag" regression analyses.

**Left. Fig 4d** replicated.

**Right.** The single-lag analysis repeated with a variance partitioning approach (To reveal the Unique contribution of Whisper L6, predictions made by [Whisper:L6, Env, Dv, GPT-2:L16, GPT-2:Word-Surp ] and [ Env, Dv, GPT-2:L16, GPT-2:Word-Surp ] were differenced, as in other analyses in the manuscript (but with a single-lag). Under this approach Whisper L6 appears to track a double humped response, which might reflect consecutive stages of prelexical and lexical processing as anticipated by a reviewer. However, care should be taken in interpreting variance partitions because stimulus features that are shared across models might be encoded at different stimulation latencies.
