# Supplementary material for: Deep-learning models reveal how context and listener attention shape electrophysiological correlates of speech-to-language transformation
Source: PLoS Comput Biol. 2024 Nov 11;20(11):e1012537. doi: 10.1371/journal.pcbi.1012537 (PMC11581396; doi:10.1371/journal.pcbi.1012537)
Supplement: Attachment — Submitted filename: plos_compbio_nov_2023.pdf [file pcbi.1012537.s002.pdf]

Review of “Context and Attention Shape Electrophysiological Correlates of Speech-to-Language Transformation”

In this study, the authors use sets of features drawn from the latent space of the WSPSR/Whisper network (Radford et al. 2022) to predict EEG data recorded while participants listened to audiobook passages in English. Using one dataset, the authors found that features drawn from the innermost layer of Whisper-base (L6) explained more of the neural response to speech than 1. the acoustic envelope and its first derivative, 2. eighty mel-scaled spectrographic features, and 3. word surprisal values estimated with GPT-2. Additionally, using a dataset in which participants had been told to listen to only one of two simultaneously-presented audiobooks, the authors find that Whisper L6 features better explain the neural response to attended speech than unattended speech. For both datasets, the electrodes best explained by the Whisper feature sets were those typically associated with acoustic processing. Based on these findings, the authors argue that they find “new EEG correlates of speech-to-language transformation”.

The application of Whisper-based feature sets to the decoding of scalp EEG data is novel. However, in the current manuscript, the authors’ claims for the significance of their results are not sufficiently supported. I have three main critiques:

1. Key aspects of the research question are inadequately developed.
2. Some analytical choices are inadequately motivated.
3. Controls are generally inadequate for the claims made.

I outline each of these critiques in more detail below, alongside approaches towards strengthening future versions of the manuscript. However, given the substantiveness of the recommended revisions at this time, I do not think that this manuscript is ready to be considered for publication in PLOS Computational Biology.

1. Key aspects of the research question are inadequately developed.

Some terms that are central to the research question of this work are left poorly defined. Foremost of these, variations on the phrase “speech-to-language transformation” are used to describe both what the human brain does and what the Whisper encoding network does. Based on these usages, it is unclear what the authors think language is. Language is a topic of intense research, and there exist many well-described proposals for how it might work, including generative, categorial, and analogical proposals, among many others. Engaging with a recognizable theory of language I think will be crucial to better targeting the research question to strengthen this manuscript.

Similarly, one of the central claims of the manuscript is that it provides evidence of a novel EEG signature of language processing. Throughout the manuscript the authors also refer to an “EEG signature of Lexical Surprisal”, “traditional N400 signatures of lexical processing”, and “signatures of word processing”. In these cases, it seems like the word *signature* is being used as a catch-all term for “brain measurement that correlates with X”. In this way, the use of the term *signature* both cheapens the very precise behavior of the N400 response and oversells the

(lack of) consensus in the field about neural correlates of lexical surprisal and lexical processing more generally. Moveover, in defaulting to a vague term like *signature*, the authors underdefine (and therefore likely undersell) their own contribution to the literature. I want more specific and precise descriptions of the significance of the results. It's easy to find a neural correlate of cognition; it's much harder to show that it appreciably improves the field's understanding of that cognitive domain. Therefore, the authors should focus more on convincing the reader that their neural correlate helps us understand something specific that is important about language.

The use of imprecise language contributes to a research question that at times seems distracted and poorly motivated. While the introduction sets up a research goal of demonstrating the superiority of learned feature sets over a specific genre of handcrafted feature sets (those involving categorical speech sound labels), this is never tested. Instead the results compare Whisper feature sets to the speech envelope, lexical surprisal, and other learned feature sets.

Since the introduction does not motivate predictions for the comparisons that are presented in the results section, predictions described in the results often seem ad hoc and poorly motivated. For example, on page 15 of the PDF the authors write, "Given the current evidence that EEG responses captured by Whisper reflect both lexical and sub-lexical structure we further examined how their timing related to responses to acoustic speech processing and language with the natural expectation that Whisper would be intermediary." Why this is a natural expectation is unexplained: If Whisper reflects both lexical and sub-lexical structure, why would its peak explanatory latency be expected to be intermediary and not double-humped?

Related to these issues, the work engages inconsistently with appropriate literature. Three areas stand out.

1. Despite the fact that the study does not involve ERPs and Whisper features are not expected to contain information that the N400 would be sensitive to, the authors appeal to literature on the N400 throughout the paper. In one such instance on the middle of PDF page 6 the authors conflate the context sensitivity of the N400 with studies interested in phonological context (i.e., di Liberto et al. 2015 and Brodbeck et al. 2015), seemingly criticizing papers researching phonetics/phonology for not adequately integrating lexicosemantic context into their analyzes. This is a baffling argument.
2. The paper starts by describing the invariance problem, but only cites Smith (1995). This is a bit odd since discussion of the invariance problem goes back at the very least to the 1960s (see Liberman et al. 1967), and Smith (1995) is not a particularly seminal paper in that literature.
3. More recent papers in the computational cognitive neuroscience of language tend to be cited as expected. However, in their results using the attention dataset, the authors write that "unlike EEG correlates of acoustic speech, the new EEG speech-to-language signature diminished when listeners ignored one speaker in favor of listening to another competing speaker". Here, the authors are contrasting their results with those of i.e., Mesgarani & Chang (2012), but seem to misstate either what Mesgarani & Chang show or what they themselves show. Notably, Mesgarani & Chang show that unattended

speech is less accurately reconstructed from neural data. Similarly, the current study also shows that Whisper features are less successful at reconstructing the neural response to unattended speech.

2. Some analytical choices are inadequately motivated.

This section covers a handful of analytical choices that lack sufficient motivation in the text.

a. In Figure 1, the significance of the bottom row of plots is unclear. At the very least, the description of the plot axes should be clearer. I understand that the color in the bottom left of the matrix plot is intended to show that some attention weights are non-zero for words prior to the current word, indicating integration of previous information into current activations. Is the model weighting itself significant in some way? If so, on what basis are certain weightings significant? It may be helpful to see some kind of legend showing the range of weight values in the plot.

b. At several points, models are claimed to yield “highly accurate predictions”, but the maximum r value in the study appears to be 0.1, even for per-electrode (non-scalp average) measurements. These r values are on par with (if not a little lower than) values reported for analyses on this very same dataset in di Liberto et al. (2015). Di Liberto et al. use simpler, handcrafted feature sets. So, on what basis are the models in this study “highly accurate”? Even if this language is simply exaggeration, I think it’s worth confronting di Liberto et al.’s results head on: do the Whisper features better explain the data at certain electrodes perhaps? or do they explain the same data as Di Liberto et al.’s handcrafted feature sets?

c. In Figure 2, the r values of the Env&Dv, Log-Mel Spectrogram, and GPT-2 word surprisal are practically identical. This is unexpected; why could this be? If I understand correctly, the Env&Dv feature set has two features per time bin, the Log-Mel Spectrogram feature set has 80 features per time bin, and the GPT-2 word surprisal model has one feature per time bin. It seems odd that 80 spectrographic features are not noticeably more informative of neural response than two acoustic correlates of loudness or one lexical statistical measure.

d. Overall for Figure 2 what I see is that the Left plot tells me that envelope and first derivative are features that Whisper does not extract by itself. The Middle plot tells me that Whisper already has log-mel spectrogram information (which is known from how Whisper is trained). The Right plot tells me that although longer range statistical/acoustic features of speech are extracted in deeper layers of Whisper, word surprisal itself is not something that Whisper extracts. Altogether, these insights characterize more about what information the Whisper feature set contains than how the brain processes language. This raises one of my biggest concerns about this study: the double black box problem.

Throughout the paper, it seems like Whisper is mostly a fancy feature engineering technique, with the unfortunate downside that we know relatively little about what in those engineered features drives their modest ability to predict brain data. All we can say from this paper is that Whisper doesn't completely recapitulate Env&Dv information, word surprisal, or GPT-2 layer 16 information. Unfortunately this poses real challenges for the significance of the results to cognitive neuroscience: are the brain data being used as evidence about the structure of the Whisper network, or are the network features telling us something about the brain? As a potential PLOS Comp Bio paper, I would hope for the latter, but in this paper I mostly see the former.

This is a hard problem in computational cognitive neuroscience right now, and it resists easy solutions. All I can recommend for now is that the revised manuscript will likely be stronger if it exploits something concretely known about the structure of Whisper and/or Whisper-derived features as a lever to explain brain data.

3. Controls are generally inadequate for the claims made.

This section contains more targeted questions about the analysis and how particular analyses are interpreted.

a. What was done to control for the fact that most of the control models have fewer parameters than the Whisper model?

b. On PDF page 10, the authors write that when fitting models for EEG responses to unattended speech, "traces of low-level acoustic speech processing remain (Broderick et al. 2018), albeit with a reduced magnitude". Poorer fit of acoustic models on the brain response to unattended speech is also reported in Mesgarani & Chang (2012). The general consensus in the literature is that attention increases the fidelity of acoustic encodings of speech. That being said: Whisper is a transformer encoder that takes purely acoustic information as input and is trained to compress that information in a way that can be most accurately used to mark word boundaries and map speech to text. Thus, the simplest explanation for the decrease in Whisper L6 performance when predicting the neural response to unattended speech is that Whisper provides a sophisticated compression of acoustic features, features which are less faithfully tracked by the brain when not attended to. In other words, Whisper is not sensitive to attention in the brain, but to acoustic features, the neural availability of which is modulated by attention.

c. Why does shuffling or averaging the Whisper L6 features improve the fit for six of the participants in Figure 4?

d. Additionally for the shuffle manipulation depicted in Figure 4, I would expect shuffling vectors to decrease model performance generally, so I would like to see that it actually

matters that the vectors are shuffled “within word” and not just any random comparably sized set of vectors getting shuffled.

e. As mentioned in Section 1 above, Figure 4 (right) is used as evidence that the Whisper feature set captures sub-lexical information intermediary to acoustics and lexical information. How does that follow from the data given? On PDF page 13, the authors write, “Consistent with EEG reflecting traces of lexical processing we found that late linguistic Whisper layers captured all variance predicted by GPT-2.” This argument suggests that two feature sets accounting for the same *amount* of the variance are accounting for the same variance, which is not in general true.

f. As mentioned in both Sections 1 and 2 above, throughout the analyses it’s unclear what the counter-hypotheses would be for many analyses. Based on the introduction and an unspecified “sub-lexical” concept introduced in Figure 4, the authors seem to want to claim that the Whisper L6 features better explain brain data than transparently categorical models of speech sounds. Why isn’t this tested head-to-head?

g. On PDF page 13, the authors write that “accuracy was greatest at 10s, suggesting that intermediate contexts – which could support extraction of semantics and syntax– are valuable”. Given the fact that Whisper is trained on exclusively acoustic information, it’s unreasonable to jump straight to the idea that 10s latencies support semantic or syntactic information. It’s far more likely that the efficacy of 10s contexts is related to cross-speaker normalization, F0 normalization across utterances within speaker, accent stabilization, or consistency of acoustic cues to word boundaries. If the authors would like to appeal to syntax or semantics in this manuscript, they should offer more evidence for its relevance.

Miscellaneous

- PDF Page 6 middle of Paragraph 1: The author is Gillis, not Gillest. (It’s correct in the bibliography)

- What is the dotted black line in figure 4 Mid-left? Just the value for the 0.5s sliding window? I think this should be mentioned explicitly somewhere.

- Also for Figure 4 (mid-left), in the figure it looks like 5s, 10s, and 20s lags are not significantly different from one another, but on PDF page 14 it says that only 10s and 20s were not significantly different from one another. What is correct?

- PDF Page 10: “listeners can home in on a single speaker” It should be ‘hone.’

- In general, all figures would be improved with lettered subfigure labels (i.e., a, b, c)

- What do attended and unattended look like per audiobook condition?

- Are signed ranks tests between Control and Whisper models reported somewhere? Did I miss them?

- PDF Page 15, Figure 5 Caption: “Wav2Vec2 and HuBERT both yielded highly accurate predictions but unlike Whisper, the inner layers were accurate.” Doesn’t Figure 5 show that inner layers of Wav2Vec2 and HuBERT are less accurate than Whisper?

- PDF Page 15: “Please note also, that GPT-2 and probably Whisper (as above) are anticipatory, and may capture brain responses at short-latency.” What is meant by this?

- I personally wouldn’t use PCA to compress the Whisper L6 feature space because it’s a pretty crude and lossy compression technique. It’s likely that the advantage of the PCA dimensions is maxing out at 10 just because the compression is poor quality. Have you tried training an autoencoder to reduce the dimensionality of the Whisper feature space?

- Supplementary Figure 5, top right: I don’t think this style plot is best for a categorical measure. At the very least, the legend should be categorical.
