# Supplementary material for: Deep-learning models reveal how context and listener attention shape electrophysiological correlates of speech-to-language transformation
Source: PLoS Comput Biol. 2024 Nov 11;20(11):e1012537. doi: 10.1371/journal.pcbi.1012537 (PMC11581396; doi:10.1371/journal.pcbi.1012537)
Supplement: Attachment — Submitted filename: eeg_speech_to_language_transformation_response1.pdf [file pcbi.1012537.s003.pdf]

Dear Dr Martin and Marinazzo,

Thank you very much for arranging for the review of our manuscript. Apologies also for the long delay in getting the revisions back, we got a late start due to a confluence of urgent commitments all popping up at once.

We have substantively revised the manuscript and have attended to all of the reviewers' comments, paying particular attention to the theoretical development the work, the control analyses and comparisons to models with interpretable annotations. We thank the reviewers for their time and believe the manuscript to be substantially improved in response to their comments.

Please find below an itemised list of our revisions. Revisions to the manuscript text are highlighted in green font (in the below responses and the re-submitted manuscript).

Thanks again for your interest in this manuscript, and we hope the revisions will be satisfactory.

Yours sincerely,

Andrew Anderson, Chris Davis, Ed Lalor

Reviewer's Responses to Questions

Comments to the Authors:
Please note here if the review is uploaded as an attachment.

Reviewer #1: Please see uploaded attachment.

Reviewer #2: Anderson etal report a range of attempts to model EEG responses to naturalistic speech (both single- and multispeaker conditions) with features extracted from off-the-shelf end-to-end deep neural networks trained to process speech sounds, particularly focusing on "whisper", a model trained to transcribe speech to text. They compare the prediction performance to a range of baseline models and find that whisper, especially its latest layers, makes the strongest contribution to predicting hold-out data, while the envelope with its 1st derivative as well as word surprisal also contribute features unavailable from whisper. They then show that these findings are specific to the responses to an attended speaker when presenting multispeaker stimuli. Lastly, they apply a range of tests to interpret the contribution of whisper, focusing on 1) comparisons to the natural language processing model GPT-2 (the last whisper layer fully accounts for GPT-2's contributions), 2) the length of the context provided to whisper (finding that a length of ~10 seconds is best suited to predict EEG responses), 3) the relevance of temporal structure within words (finding that shuffling whisper representations within words harms prediction performance and that the contribution of whisper is thus partly sub-lexical), 4) contributions of individual lags (finding that early lags seem beneficial for acoustic models, mid-range lags seem beneficial for

sublexical features such as whisper and long lags seem beneficial for word surprisal effects) and 5) a comparison against self-supervised speech models, finding that none of them can predict EEG better than whisper.

This impressive set of results is paired with an extensive set of visualisations, supplementary figures and highly detailed descriptions of the methods. The authors mainly use careful and suitable analyses and mostly position their research well within the current, quickly developing landscape of related publications. In sum, the authors make it very hard for me to find aspects that could be done differently.

Thanks for your careful attention to the manuscript and the flattering comments !

I will here list the result of such efforts:

Title (and abstract)

Within the context of transformer models, it could be helpful to rethink the use of the word "attention" in the title and the abstract (where "listener-attention" is used). I am not a computer scientist, but I feel that this work seems to be relevant beyond the field of cognitive neuroscience and might very well be cited in computer science circles. If the authors manage to find a way to rearrange the title to make it clear that the attention they are referring to is not that of their model, but that of the listeners (I assume this refers to the results in figure 3), this could help with a quick interpretation of the results from skimming its title in reference sections of citing papers to come.

Thanks for the suggestion, we had wondered about this too. We've added in a "listener" and a "deep-learning models" to the title to help clarify.

Deep-Learning Models Reveal Context and Listener Attention Shape Electrophysiological Correlates of Speech-to-Language Transformation

Abstract

It could be helpful to point out here what the optimisation objective of whisper is (textual annotation, as far as I understand).

Further, I am unsure whether the use of "accurate" is justified -- after all, the models only explain very small amounts of variance or correlation, and when the audience reads that these models are performing accurately, they might easily get the wrong impression. Critically, I do not think it is necessary to communicate how impressive these results are.

That's fair, we've expunged remarks suggesting high accuracy from the manuscript, and mentioned the speech-to-text in the new abstract, which is revised according to this comment and other reviewer comments, as below (changes in green).

Abstract To transform continuous speech into words, the human brain must resolve variability across utterances in intonation, speech rate, volume, accents and so on. A promising approach to explaining this process has been to model electroencephalogram (EEG) recordings of brain responses to speech. Contemporary models typically invoke context invariant speech categories (e.g. phonemes) as an intermediary representational stage between sounds and words. However, such models may not capture the complete picture because they do not model the brain mechanism that categorizes sounds and consequently may overlook associated neural representations. By providing end-to-end accounts of speech-to-text transformation, new deep-learning systems could enable more complete brain models. We model EEG recordings of audiobook comprehension with the deep-learning speech recognition system Whisper. We find that (1) Whisper provides a self-contained EEG model of an intermediary representational stage that reflects elements of prelexical and lexical representation and prediction; (2) EEG modeling is more accurate when informed by 5-10s of speech context, which traditional context invariant categorical models do not encode; (3) Deep Whisper layers encoding linguistic structure were more accurate EEG models of selectively attended speech in two-speaker "cocktail party" listening conditions than early layers encoding acoustics. No such layer depth advantage was observed for unattended speech, consistent with the brain's more superficial level of linguistic processing.

Introduction

1) The present work has similarities to that of Millet and Vaidya. These papers are cited at a later stage, however, I think they could already be cited in the introduction, together with the work of Li et al., nature neuroscience 2023 (https://www.nature.com/articles/s41593-023-01468-4) and potentially the work of Boos et al., NeuroImage 2021 (https://www.sciencedirect.com/science/article/pii/S1053811921003839) which also attempt to model responses to speech with models starting from acoustic features.

Millet, Vaidya and Li are now cited in the Introduction. We had trouble slotting Boos into the natural flow though, given that much of its focus is on component modeling as opposed to modeling across layers.

To characterize the correlation between EEG and Whisper we first examined how EEG predictions vary across model layers, as has been examined with language models in fMRI, MEG or ECoG (Jain and Huth, 2018; Toneva and Wehbe, 2019, Sun et al. 2020, Anderson et al. 2021, Schrimpf et al. 2021, Millet et al. 2021, Caucheteux et al. 2023, Goldstein et al. 2023, Antonello et al. 2024), and more recently speech models (Millet et al. 2022, Vaidya et al. 2022, Li et al. 2023, Goldstein et al. 2023, Antonello et al. 2024). We analyzed both audiobook comprehension EEG recordings made in: (1) single-speaker listening conditions, and (2) an

experimental “cocktail-party” scenario where participants listened to two concurrent audiobooks but paid attention to only one (O’Sullivan et al. 2014)….

2) "such contextual information is not present in purely categorical speech models" -- what exactly is meant by "categorical" here? It seems slightly ambiguous, as I don't see a theoretical reason why categorical models couldn't also include contextual information.

We agree that this was vague. What we really meant was context invariant models. We’ve explicitly added “context invariant” in the text when this is what we mean. This appears in the abstract in the earlier response, and also in the first Discussion paragraph, as below.

The current study has revealed electrophysiological correlates of the linguistic transformation of heard speech using the end-to-end speech recognition model Whisper. This addresses a limitation of previous work that has typically relied upon hand-crafted context invariant categorical speech units such as phonemes to capture an intermediary phase between sound and words, and thereby neglected to model a mechanism that maps sounds to categories, and potentially also the representations invoked in this mapping.

Results

0) In either the introduction or the results section, a clear description of the optimisation objective of whisper should be provided. What does it ultimately predict? Is the loss a cross-entropy over words? Does it build a vector representation of such words in a similar way as word2vec, or does it only model a discrete probability distribution? This would also make it easier to process the later "... that deeper and *more linguistic* Whisper layers were the strongest EEG predictors.". In the methods section, we find "transforms spectral speech features into word representations", but we do not learn about the nature of these word representations.

We agree our initial attempt may not have hit the mark. We’ve had another go at concisely giving the reader a better sense of Whisper at an early stage in the Introduction.

By modeling speech recognition end-to-end from audio to words, with human-like accuracy, recent deep artificial neural networks such as Whisper (Radford et al. 2022) present opportunities to alleviate concern (1) above and potentially provide a new window on speech comprehension in the brain. Critically, different to categorical speech models, intermediary representations within Whisper are a learned function of the audio spectrogram, that was optimized to reproduce speech transcriptions made by human annotators. Thus, Whisper might not only discover intermediary phoneme representations but also learn how to exploit phonetic and lexical context in service of speech recognition, which in turn might model new and/or known electrophysiological correlates of phonetic and lexical processing in natural speech comprehension (e.g. di Liberto et al. 2015, Daube et al. 2019, Brodbeck et al. 2018, Broderick et al. 2018, Broderick et al. 2019, Heilbron et al. 2022). Indeed, recent empirical studies (Kloots

and Zuidema 2024, Pouw et at. 2024) of the self-supervised speech model Wav2Vec2 (Baevski et al. 2020) have demonstrated sensitivity to phonological context and lexical knowledge, and more generally a range of different speech models have been observed to encode phonemes (Martin et al. 2023) and syntax and semantics to a degree (Pasad et al. 2024).

Operationally, Whisper turns continuous audio speech into categorical word units via a succession of intermediary transformations that take place within an “Encoder-Decoder” Transformer architecture (Vaswani et al. 2017). The Encoder module prepares input speech spectrograms for word decoding. This is achieved by re-representing each spectrogram timeframe as a “contextualized” weighted average of itself and all other time frames within a 30s window. The contextualization process is repeated across multiple intermediate layers, each feeding forward into the next. The output of the final layer, which is the output of the entire Encoder module is a time-series of contextualized speech vectors that are fed as one 30s chunk into the Decoder. The Decoder is also a multilayer feed-forward Transformer network, which then transcribes the encoded speech into a series of discrete word units in the same or a different language. This proceeds as an iterative process, with the decoder predicting the identity of the next word based on the encoded speech and any words it has previously decoded. Thus, the decoder closely resembles next-word-prediction language models such as GPT-2 (Radford et al. 2018), but with the additional access to contextually encoded speech.

Here, we model audiobook speech EEG recordings with Whisper’s Encoder module, with an eye to identifying whether Whisper affords a predictive advantage over traditional acoustic measures, which we find, and then characterizing what underpins this advantage. We based our EEG analyses solely on Whisper’s Encoder on the assumption that it plays the key role in transforming audio speech into a linguistic form to help the Decoder to predict word identity, otherwise the Encoder would be redundant. We further assumed that Encoder representations would become more linguistic with layer depth due to the feedforward architecture and contextualization process (see Methods for more details on these assumptions). However, the degree to which the learned linguistic forms reflect sub-words, words, or even semantics to successfully interface with the word decoder, and which of these features contribute to EEG models requires further investigation to estimate, which we undertook. To ease descriptions in the forthcoming text, we refer to the transformation performed by Whisper’s Encoder as a speech-to-language transformation – with the proviso that language is ambiguously defined – and the conclusion we finally reach is that Whisper learns a mixture of sub-word, word structure, in part reflecting lexical predictions, and this helps to model EEG.

1) "both in natural listening conditions and when speech is paid attention to or not" -- to me, both of these conditions seem "natural". Maybe one could be called "passive" rather than natural?

Thanks, we’ve changed natural listening conditions to single-speaker listening conditions to circumvent this issue.

2) Reporting the correlation values, but then referring to squared correlation values which "double" seems confusing to me. Might it make more sense to just stick to the correlation values for this section?

We've just stuck with presenting the correlation coefficients and have deleted the "doubling" statement.

3) "The set of correlation coefficients" -- Which set? This refers to the slope over layers?

Yup. Please see below.

The Mean±SEM Spearman correlation coefficient between prediction accuracy and layer depth (0 to 7) across participants was 0.77±0.07. The set of layer depth vs prediction accuracy correlation coefficients (for the 19 participants) were significantly greater than 0 (Signed-rank Z=3.7924, p=1.5e-4, n=19, 2-tail). This provided evidence that deeper and more linguistic Whisper layers were the strongest EEG predictors.

4) " ... but not the Spectrogram (Whisper's input) uniquely predicted elements of the EEG signal" -- please refer the reader to the corresponding part of figure 2.

We've added the text below.

To identify which Control models complemented Whisper, we evaluated whether Union prediction accuracies were greater than constituent Whisper layers (Signed ranks tests, one-tail, 19 subjects). This revealed that both Env&Dv and Lexical Surprisal (**Figure 2** Top Left and Top Right), but not the Spectrogram (Whisper's input) uniquely predicted elements of the EEG signal (**Figure 2** Top Middle). In sum, these results provide evidence that EEG strongly reflects the transformation of speech to language encoded by Whisper.

5) Figure 2: I might be wrong, but this colourmap looks to me like jet, which has been shown to be suboptimal. A perceptually linear colourmap might be better suited to communicate these results (e.g. viridis, or "turbo" if the authors want to stick to a rainbow scheme).

You're right it is Jet. We hope you'll excuse us for not changing this. On viewing the similarities in appearance between Turbo (Left below) and Jet (Right below), we felt that the cost-vs-benefit of regenerating and re-collating all the multi-piece figures again fell in favour of sticking with Jet. However, in the future we'll use Turbo from the onset.

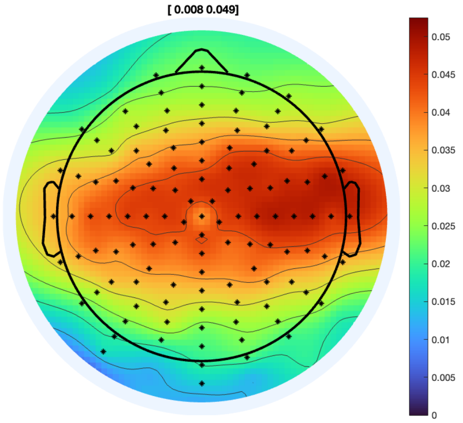

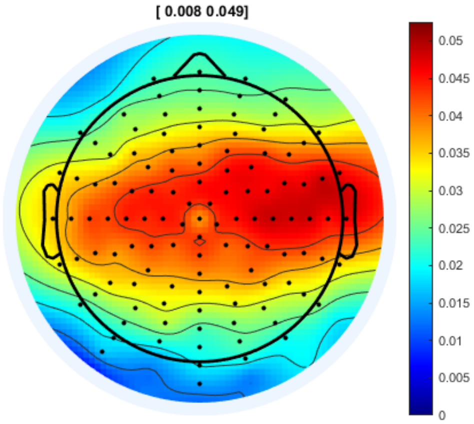

6) The section reporting results from multispeaker studies could also cite the work by Fiedler et al., NeuroImage 2019 (https://www.sciencedirect.com/science/article/pii/S1053811918320299).

Now cited.

7) "Model features were offset by a single lag" -- this seemed somewhat ambiguous to me -- I assume the authors mean that only a single lag of the features were used to predict EEG rather than the full set. Maybe that is just my reading, but it could potentially be helpful to others to make this more explicit.

You're right there, we've tried to make this clearer, below.

Given the current evidence that EEG responses captured by Whisper reflect lexical and sub-lexical structure we further examined how their timing related to acoustic speech processing and language with the expectation that Whisper would be either intermediary (reflecting a sub-lexical/lexical feature mixture) or would separably reflect both. To explore this, we ran a set of analyses where only a single time-lag of model features was used to predict EEG, rather than all time-lags at once as in our other analyses. Single lags were within the range [0 to 750ms] in 1/32s steps. We reasoned that prediction accuracies derived from different lags would provide an estimate of the EEG response time-delay associated with each model. We were especially interested to see if such an analysis would show a single peak at intermediary lags (indicating an intermediary sub-lexical/lexical feature mixture) or whether it would show a double peak (suggesting separable indexing of sub-lexical and lexical features).

Furthermore, this analysis could in principle be carried out in the same style as the other analyses employing variance decomposition, or alternatively the authors could here train the models by excluding the lag in question and then observing whether the performance decreases relative to the full model. Including an invididual lag doesn't tell us whether this particular lag uniquely carried

predictive power, and therefore complicates the interpretation.

Fair point. We'd erred towards a single model analysis, because we found it easier to interpret and single lag variance partitions potentially confusing (for the reasons stated below). Nonetheless, we've conducted the variance decomposition approach and included the outcome in supplementary materials. The entire section (part above) and new supplementary figure are below.

Interpretation: Whisper best predicts EEG responses that are intermediary between speech acoustics and language

Given the current evidence that EEG responses captured by Whisper reflect lexical and sub-lexical structure we further examined how their timing related to acoustic speech processing and language with the expectation that Whisper would be either intermediary (reflecting a sub-lexical/lexical feature mixture) or would separably reflect both. To explore this, we ran a set of analyses where only a single time-lag of model features was used to predict EEG, rather than all time-lags at once as in our other analyses. Single lags were within the range [0 to 750ms] in 1/32s steps. We reasoned that prediction accuracies derived from different lags would provide an estimate of the EEG response time-delay associated with each model. We were especially interested to see if such an analysis would show a single peak at intermediary lags (indicating an intermediary sub-lexical/lexical feature mixture) or whether it would show a double peak (suggesting separable indexing of sub-lexical and lexical features). Model-to-EEG mappings were fit on isolated models without variance partitioning to simplify analyses, and because stimulus features that are shared across models might be encoded at different stimulation latencies. In turn, this may exaggerate estimates of models' unique predictive contribution (NB the multi-lag regression analyses presented in the other results account for this). Therefore, for completeness we include a single-lag variance partitioning analysis in **Supplementary Figure 12**.

Figure 4d illustrates the EEG response timings preferentially predicted by the different models (scalp-average prediction accuracies). Whisper preferentially predicted EEG at a time delay of 63ms which was intermediary between the speech envelope (31ms) and the language model (GPT-2 L16 activation, 125ms) and word surprisal (406ms), however Whisper's prediction accuracies were also comparatively high across the 400ms time span. This suggests different L6 features may have value for predicting different stages in conversion from sounds to words, and in particular early prelexical representation. A secondary observation was that GPT-2's L16's prediction accuracy profile was doubled humped across response lags, with the second (weaker) peak at 563ms. We speculate the GPT-2 double hump reflects EEG responses associated with consecutive words e.g. the language model at word n both predicts the EEG response to word n and also n+1 (albeit with reduced accuracy).

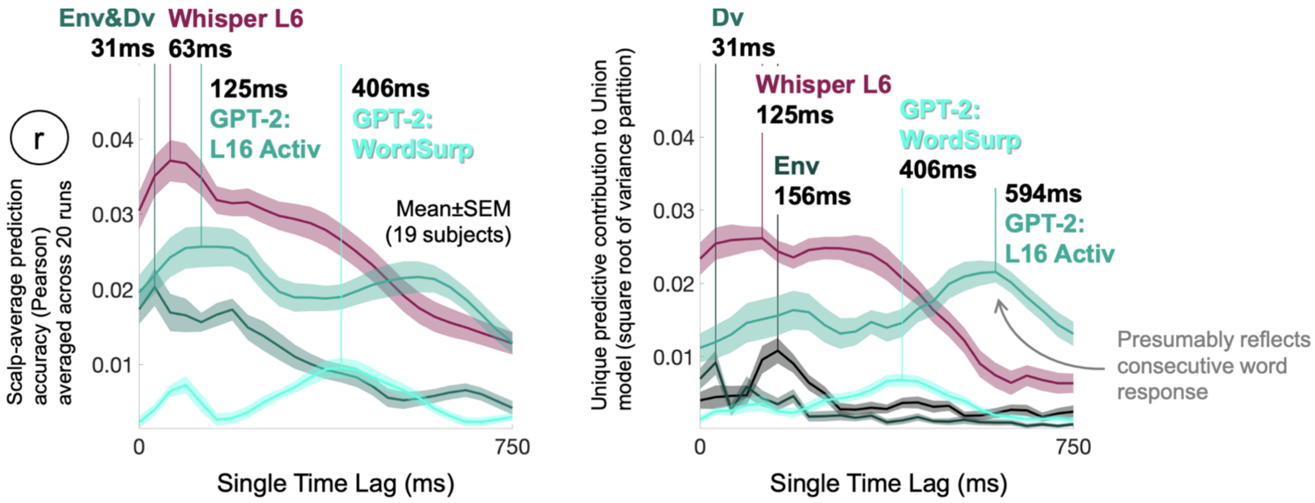

Supplementary Figure 12 (Figure 4 Companion). To explore how the relative timing of EEG responses predicted by Whisper compared to the speech envelope and language model, we ran a battery of "single time lag" regression analyses.

Left. Figure 4d replicated.

Right. The single-lag analysis repeated with a variance partitioning approach (To reveal the Unique contribution of Whisper L6, predictions made by [Whisper:L6, Env, Dv, GPT-2:L16, GPT-2:Word-Surp] and [Env, Dv, GPT-2:L16, GPT-2:Word-Surp] were differenced, as in other analyses in the manuscript (but with a single-lag). Under this approach Whisper L6 appears to track a double humped response, which might reflect consecutive stages of prelexical and lexical processing as anticipated by a reviewer. However, care should be taken in interpreting variance partitions because stimulus features that are shared across models might be encoded at different stimulation latencies.

Discussion

The authors wonder why longer context windows are disadvantageous. It would be interesting to see here how different context lengths affect the performance of whisper on its native optimisation objective -- are 30 seconds more helpful to transcribe than 20 seconds? Ideally, the authors would have access to whisper models trained on different context lengths to generate a 2D representation in the classic style of Yamins et al., 2014 PNAS (Figure 1) or Schrimpf et al., PNAS 2021 (Figure 3). Since this might however be prohibitively cumbersome, the authors might discuss this issue.

Thanks for the suggestion. Please see below:

<snip> …we examined the impact of limiting Whisper's opportunity to form predictions, by limiting speech context. Despite our anticipation that EEG prediction accuracy might monotonically improve or asymptote with longer contexts (up to the 30s max), we found 5-10s

context to be most accurate (~20% extra variance predicted than .5s). Critically the 5-10s contextual advantage provides evidence that EEG encodes information across multiword speech contexts, and thereby questions the completeness of categorical speech models that are context invariant. However, it remains unclear why 5-10s speech is advantageous and future work will be needed to characterize EEG correlates of speech contextualization at lexical and prelexical levels. One approach could be examining how representations in speech models vary in the presence/absence of linguistic contexts that do/don’t make upcoming words and sounds predictable (e.g. Pouw et al. 2024) and then testing whether model differences capture corresponding differences in electrophysiological responses to the predictable/unpredictable stimuli.

Another potentially revealing approach could be to examine how speech model transcription performance varies when models are optimized on different context lengths, and how this relates to EEG modeling accuracy. Although experimentally retraining Whisper was beyond the scope and means of the current study, we note that Jain and Huth (2018) ran a comparative language modeling analysis evaluated on fMRI data. In training an LSTM language model on next-word-prediction, they observed negligible word prediction benefit to training with contexts of more than 20 words, and that fMRI prediction accuracy also began to asymptote when models neared a 20-word context. On face value, this 20 word context is consistent with 10s speech, given that one would expect at least 20 words to be spoken in 10s (assuming 120-200 words are spoken per minute Crystal et al., 1990, Liberman et al., 1967). However, given that people don’t altogether forget what they heard 10s ago, large language models are now routinely trained on thousands of word contexts (Touvron et al. 2023), and large language and speech models can improve fMRI modeling accuracy (Schrimpf et al. 2021, Antonello et al. 2024), future work will be required to explore the nature of this correspondence.

Typos:

- responses reflect abstract speech representation as opposed to ... -- in my view, "representation" lacks an s

Done

- This enabled the analyses to be standardized to be have exactly -- "have" should go

done

- was that we discontinued the using Log-Mel ... -- "the" should go

done

- EEG Prediction accuracies derived from attended speech models resembled ... -- "Prediction" shouldn't be capitalized

done

- MEG, ECoG and EEG (Goldsetin et al., 2022) -- should be "Goldstein"?

done

Thanks again for your comments, interest and the time that went into this review.

Reviewer #3: Overall:
The authors present a study investigating the EEG correlates of speech-to-language transformations facilitated by pre-trained deep learning model Whisper. The novelty of this study is in using EEG (instead of fMRI or ECoG) and then investigating the effects of temporal context and attention on predictions (temporal context, in particular, is something other studies have begun looking into, albeit with different models and input modality data).

The study describes the results and interpretations clearly, and the addition of the cocktail party dataset analysis is particularly great in showing that predictions are not resulting from low level acoustic processing. I have no concerns about the analyses themselves.

Thanks for your interest and encouragement.

My concerns are primarily regarding clarity and contextualization. I have major concerns with the introduction and discussion on these issues. Improving on these fronts will, I believe, not only make this report more easily understandable to an audience that either isn't as familiar with speech and language or isn't as familiar with correlating deep learning/LLMs with neural activity, but will also strengthen the impact of the current study.

That's fair, it was tricky to get a good balance here. We've had a go at fixing this up.

Major:
1- Intro first paragraph: The authors state that categorical models (presumably they are referencing things like acoustic-phonetic STRFs) "cannot capture associated brain activity." Of course a STRF is not providing a mechanism, but this statement seems naive/misleading as these models are, by definition, fit to reconstruct brain activity. So by definition, it appears to me that they _do_ capture brain activity, they just do not give a comprehensive mechanism. The authors should either clarify why this statement is still true or edit it accordingly.

We've amended the abstract as below:

Contemporary models typically invoke context invariant speech categories (e.g. phonemes) as an intermediary representational stage between sounds and words. However, such models may not capture the complete picture because they do not model the brain mechanism that categorizes sounds and consequently may overlook associated neural representations.

As well as the introduction.

By enabling temporally precise estimates of brain activity, electrophysiological measures such as scalp EEG have provided evidence that the brain transforms natural continuous speech into words as a cascading process, with abstract categorical speech units such as phonemes or their articulatory features serving as intermediary pre-lexical representations (di Liberto et al. 2015, and more recently Gilles et al. 2023). To support this, researchers have typically revealed how EEG models of speech comprehension are improved when representing the speech stimulus as a time-series of categorical phoneme feature vectors in addition to the audio signal. However, the strength of this evidence has been critiqued (Daube et al. 2019) because: (1) Categorical speech models typically do not specify the computational mechanism that categorizes variable speech sounds, nor how sub-word representations are derived from audio data (because categories are manually configured by experimenters). They therefore may miss out on key transformational stages.

2- Intro first paragraph statement #2: I think more context is needed on why modelling information not available (such as phonemes) to participants' brains is limiting. Do the authors believe that phonemes (or whichever speech category) are not part of any intermediate representation? Or do the authors mean to say that this doesn't give the full picture? The way this is written, it is unclear to me which, and I think that (at least a good portion of) literature supports that phonemes are a useful representation at some level.

We've sought to clarify this throughout the manuscript. The argument we had intended to convey was modelling phonemes doesn't provide the complete picture. This should come out in our above responses. There's another statement of a similar ilk in the Discussion, as below.

The current study has revealed electrophysiological correlates of the linguistic transformation of heard speech using the end-to-end speech recognition model Whisper. This addresses a limitation of previous work that has typically relied upon hand-crafted context invariant categorical speech units such as phonemes to capture an intermediary phase between sound and words, and thereby neglected to model a mechanism that maps sounds to categories, and potentially also the representations invoked in this mapping. The current results suggest there is benefit to modeling EEG with a more complete model and suggest that this approach reveals correlates of a contextualized transformation that reflects both prelexical and lexical representation and predictive processing, and which cannot be comprehensively modeled by context invariant categorical approaches (by definition).

3- Throughout the paper (but first mentioned in the intro third paragraph), later layers are referred to as "more linguistic." This is too vague and I think would benefit from either referencing literature that explicitly tests this, or at the very least some other qualitative explanation. There is some mention of these types of analyses (done by other groups) later in the discussion, but I think it's important that this is explained up front as much of the analysis is based on this understanding.

Thanks for pointing this out. We've had a go at rewriting the Intro to clarify some of this, and pointing the reader towards the Methods where the assumptions about whisper representations are fleshed out in more detail.

By modeling speech recognition end-to-end from audio to words, with human-like accuracy, recent deep artificial neural networks such as Whisper (Radford et al. 2022) present opportunities to alleviate concern (1) above and potentially provide a new window on speech comprehension in the brain. Critically, different to categorical speech models, intermediary representations within Whisper are a learned function of the audio spectrogram, that was optimized to reproduce speech transcriptions made by human annotators. Thus, Whisper might not only discover intermediary phoneme representations but also learn how to exploit phonetic and lexical context in service of speech recognition, which in turn might model new and/or known electrophysiological correlates of phonetic and lexical processing in natural speech comprehension (e.g. di Liberto et al. 2015, Daube et al. 2019, Brodbeck et al. 2018, Broderick et al. 2018, Broderick et al. 2019, Heilbron et al. 2022). Indeed, recent empirical studies (Kloots and Zuidema 2024, Pouw et at. 2024) of the self-supervised speech model Wav2Vec2 (Baevski et al. 2020) have demonstrated sensitivity to phonological context and lexical knowledge, and more generally a range of different speech models have been observed to encode phonemes (Martin et al. 2023) and syntax and semantics to a degree (Pasad et al. 2024).

Operationally, Whisper turns continuous audio speech into categorical word units via a succession of intermediary transformations that take place within an "Encoder-Decoder" Transformer architecture (Vaswani et al. 2017). The Encoder module prepares input speech spectrograms for word decoding. This is achieved by re-representing each spectrogram timeframe as a "contextualized" weighted average of itself and all other time frames within a 30s window. The contextualization process is repeated across multiple intermediate layers, each feeding forward into the next. The output of the final layer, which is the output of the entire Encoder module is a time-series of contextualized speech vectors that are fed as one 30s chunk into the Decoder. The Decoder is also a multilayer feed-forward Transformer network, which then transcribes the encoded speech into a series of discrete word units in the same or a different language. This proceeds as an iterative process, with the decoder predicting the identity of the next word based on the encoded speech and any words it has previously decoded. Thus, the decoder closely resembles next-word-prediction language models such as GPT-2 (Radford et al. 2018), but with the additional access to contextually encoded speech.

Here, we model audiobook speech EEG recordings with Whisper's Encoder module, with an eye to identifying whether Whisper affords a predictive advantage over traditional acoustic measures, which we find, and then characterizing what underpins this advantage. We based our EEG analyses solely on Whisper's Encoder on the assumption that it plays the key role in transforming audio speech into a linguistic form to help the Decoder to predict word identity, otherwise the Encoder would be redundant. We further assumed that Encoder representations would become more linguistic with layer depth due to the feedforward architecture and contextualization process (see Methods for more details on these assumptions). However, the degree to which the learned linguistic forms reflect sub-words, words, or even semantics to successfully interface with the word decoder, and which of these features contribute to EEG models requires further investigation to estimate, which we undertook. To ease descriptions in

the forthcoming text, we refer to the transformation performed by Whisper's Encoder as a speech-to-language transformation – with the proviso that language is ambiguously defined – and the conclusion we finally reach is that Whisper learns a mixture of sub-word, word structure, in part reflecting lexical predictions, and this helps to model EEG.

To characterize the correlation between EEG and Whisper we first examined how EEG predictions vary across model layers, as has been examined with language models in fMRI, MEG or ECoG (Jain and Huth, 2018; Toneva and Wehbe, 2019, Sun et al. 2020, Anderson et al. 2021, Schrimpf et al. 2021, Millet et al. 2021, Caucheteux et al. 2023, Goldstein et al. 2023, Antonello et al. 2024), and more recently speech models (Millet et al. 2022, Vaidya et al. 2022, Li et al. 2023, Goldstein et al. 2023, Antonello et al. 2024). We analyzed both audiobook comprehension EEG recordings made in: (1) single-speaker listening conditions, and (2) an experimental "cocktail-party" scenario where participants listened to two concurrent audiobooks but paid attention to only one (O'Sullivan et al. 2014). Extrapolating from Broderick et al.'s (2018) finding that correlates of lexical processing selectively reflect only the attended audiobook, we hypothesized the same would be true for deeper-more linguistic Whisper layers, whereas correlates of lower-level acoustics would remain for both attended and unattended speech, albeit to different degrees (Mesgarani and Chang 2012, Ding and Simon 2012, O'Sullivan et al. 2014, Fiedler et al. 2019). To estimate which features of Whisper drove EEG prediction we performed comparative analyses with a pure language model (GPT-2, Radford et al. 2018) and also two self-supervised speech models (Wav2Vec2, Baevski et al. 2020 and HuBERT Hsu et al. 2021) which appear to induce aspects of lexical semantics without access to text annotations (Pasad et al., 2021; Vaidya et al. 2022). To probe for EEG correlates of subword representation we tested whether shuffling the order of Whisper vectors within words disrupted modeling accuracy. To establish that EEG responses reflect Whisper's contextualized speech transformations, we tested whether limiting Whisper's access to context compromised modeling accuracy, as has been studied in language and fMRI (Jain and Huth 2018).

4- Intro third paragraph last sentence: this sounds somewhat circular, that they paid attention to one speaker and thus that speaker is represented? I believe the authors are pointing out that purely acoustic representations could be present for both, but only higher level representations exist for the one being attended to. This would benefit from references that suggest that, so that it's clearer to the reader that not finding this is novel.

Thanks, we've now changed this to the below.

Extrapolating from Broderick et al.'s (2018) finding that correlates of lexical processing selectively reflect only the attended audiobook, we hypothesized the same would be true for deeper-more linguistic Whisper layers, whereas correlates of lower-level acoustics would remain for both attended and unattended speech, albeit to different degrees (Mesgarani and Chang 2012, Ding and Simon 2012, O'Sullivan et al. 2014, Fiedler et al. 2019).

5- Overall, I find the introduction extremely lacking in providing context of the field. There are no

references to other efforts using deep learning models, which would motivate their use (references to these only come towards the end of the discussion). The authors may find the following review paper on this subject helpful in addition to the many other studies that they do cite (at the very end of the discussion): Jain 2023 https://doi.org/10.1162/nol_a_00101. Having at least a paragraph of discussion on the advantages (or disadvantages) of these approaches would benefit the reader. I also suggest that the authors use this as an opportunity to be specific about how these types of studies can address the called-out limitations of categorical models. This can then be linked to the discussion about how this particular study was able to address some of these limitations and how further work may continue to address remaining gaps.

We hope the revisions in response to point 3 above have already helped to embed the Introduction in the wider literature.

6- Related to the previous comment, there is also little reference to other studies investigating the effects of temporal context (e.g. another LM-based brain study Jain, Huth 2018 https://proceedings.neurips.cc/paper_files/paper/2018/hash/f471223d1a1614b58a7dc45c9d01df19-Abstract.html) and attention. In particular, the Jain 2018 study is only mentioned vaguely in the discussion along with other papers looking at using LLMs to investigate brain activity. Given that it seems this paper and the Jain study share similar methods/goals, it should be introduced and discussed (e.g. where do these studies differ? Do the results corroborate each other?). It may be that the authors are trying to stick to studies in EEG, which I understand, but since modelling brain activity with deep learning models is so cross-modal (e.g. fMRI, EEG, ECoG studies all coming out), I think this needs to be introduced.

That's true, I hope the below coverage of Jain & Huth in the Discussion presents a better consideration of those findings.

[snip]…. we examined the impact of limiting Whisper's opportunity to form predictions, by limiting speech context. Despite our anticipation that EEG prediction accuracy might monotonically improve or asymptote with longer contexts (up to the 30s max), we found 5-10s context to be most accurate (~20% extra variance predicted than .5s). Critically the 5-10s contextual advantage provides evidence that EEG encodes information across multiword speech contexts, and thereby questions the completeness of categorical speech models that are context invariant. However, it remains unclear why 5-10s speech is advantageous and future work will be needed to characterize EEG correlates of speech contextualization at lexical and prelexical levels. One approach could be examining how representations in speech models vary in the presence/absence of linguistic contexts that do/don't make upcoming words and sounds predictable (e.g. Pouw et al. 2024) and then testing whether model differences capture corresponding differences in electrophysiological responses to the predictable/unpredictable stimuli.

Another potentially revealing approach could be to examine how speech model transcription performance varies when models are optimized on different context lengths, and how this relates

to EEG modeling accuracy. Although experimentally retraining Whisper was beyond the scope and means of the current study, we note that Jain and Huth (2018) ran a comparative language modeling analysis evaluated on fMRI data. In training an LSTM language model on next-word-prediction, they observed negligible word prediction benefit to training with contexts of more than 20 words, and that fMRI prediction accuracy also began to asymptote when models neared a 20-word context. On face value, this 20 word context is consistent with 10s speech, given that one would expect at least 20 words to be spoken in 10s (assuming 120-200 words are spoken per minute Crystal et al., 1990, Liberman et al., 1967). However, given that people don't altogether forget what they heard 10s ago, large language models are now routinely trained on thousands of word contexts (Touvron et al. 2023), and large language and speech models can improve fMRI modeling accuracy (Schrimpf et al. 2021, Antonello et al. 2024), future work will be required to explore the nature of this correspondence.

7- Throughout the paper (such as in the introduction, the last paragraph of "Natural Speech EEG Recordings Strongly Reflect the Linguistic Transformation of Acoustic Speech", and first paragraph of "Interpretation: EEG and Deep Whisper Layers Also Partially Reflect Sub-Lexical Structure"), the authors refer to the concept that the temporal lobe is thought to only process acoustic/spectral features, but I think this is greatly oversimplified. For example, I don't think many people would say that the STG only processes spectrograms or even just acoustic-phonetic categories (e.g. phonemes). In fact, research from various modalities suggests that things like semantics (e.g. Huth 2016 https://www.nature.com/articles/nature17637) and temporal integration across sub-word categories may be represented here (e.g., this review covers both "sound" information represented in the STG but also points to how the STG may be temporally integrating these types of representations Yi*, Leonard*, Chang Neuron review https://doi.org/10.1016/j.neuron.2019.04.023). I can understand if the authors are referencing low level auditory areas only or only criticizing linear approaches that would be best suited for low-level features, but as it is currently described, this seems to be oversimplified.

Sorry for the confusion, these statements seem to have come out wrong and had been intended to apply to bilateral scalp EEG electrodes not the temporal lobe per se. We've sought to delete statements that could be open to misinterpretation (e.g. replacing bilateral temporal regions with bilateral scalp electrodes). E.g. ...

Because the bilateral scalp electrodes best captured by Whisper (**Figure 2/3**) are typically considered to reflect low-level speech acoustics and/or categorical speech units (Di Liberto et al. 2015).

8- In the discussion first paragraph, the authors again point out the disadvantage of linear categorical models that do not model the mechanism. However, I don't see how the authors have necessarily done this either. Their analyses do suggest that representations in Whisper may be represented in the brain, but they don't analyze the computations in the model or how the brain computes this. Given that, it seems inappropriate to rely on this point for why this paper is novel. I

think it would be better to focus on how this study, by using Whisper and the various analyses, are able to probe certain features that these categorical models do not (like the temporal context), which would be important for elucidating exact mechanisms.

We've tried to restructure these arguments to not give the impression that we think we are discovering the brain's computational implementation. We hope this has already come through in our earlier responses – but in a nutshell the argument we seek to convey is that modeling an end-to-end sound categorization mechanism may discover useful representations to predict brain activity, and it turns out that knowing speech context seems to contribute to these useful representations.

9- Given that the authors note that Whisper's representations might be largely semantic, it seems odd that in the discussion, there's very little mention of the wide breadth of semantic work (e.g. work from Alex Huth's lab in particular). For example, I was left wondering whether the semantic maps found in those papers would overlap at all with the findings of this paper. Of course there is a difference in spatial resolution, but it seems that at least a sentence addressing this would place this work better in the context of other brain studies looking at representations related to the speech-to-language transformation.

We've reduced the emphasis on semantics a little, given our GPT-2 comparison is predictive lexical representation (not explicitly semantics) and the concerns of another reviewer, but we've suggested how the current results might cohere with other contemporary work, including Alex Huth and co. – as below.

To test EEG data for correlates of sub-word content we disrupted the within-word temporal structure of Whisper's final layer which we reasoned should have little effect on EEG prediction if it was driven by single code word representations. The findings were consistent with EEG additionally reflecting sub-word structure, as was evidenced by a modest reduction in EEG prediction accuracy in most participants. However, because EEG has low anatomical resolution, the degree to which this word/sub-word composite reflects intermediary part-speech part-language representational states in the brain, as opposed to a blurred sampling of distinct speech and language-selective neural populations was unclear. More anatomically precise fMRI (Antonello et al. 2024, Millet et al. 2022, Vaidya et al. 2022) and ECoG (Goldtstein et al. 2023, Li et al. 2023) studies suggest that the current EEG correlates of prelexical representation may stem from auditory cortices and in particular Superior Temporal Gyrus where intracranial electrodes are especially sensitive to contextualized phone and syllable representations from HuBERT L10 (Li et al. 2023, see also **Figure 5**). fMRI correlates of language models, and/or candidate lexical representations in speech models have typically been observed to radiate out from auditory cortex over lateral and posterior temporal zones to parietal and inferior frontal cortices (Caucheteux et al. 2022, Millet et al. 2022, Vaidya et al. 2022, Antonello et al. 2024) and presumably some of these regions contribute to the current predictive overlap between Whisper and GPT-2, and potentially also lexical surprisal.

10- One key question throughout the paper for me was whether the authors believe Whisper is the best model for this or not. For example, if others want to pursue this type of work, is Whisper the only option? I think the authors do a good job of explaining the difference between GPT-2 and Whisper (difference in inputs and training) and highlighting other models (like Wav2Vec2 and HuBERT), but a note on whether any should be used over another would be helpful. Relatedly, if there was a new LLM/deep learning model to come out, how would one decide whether it was useful to use for looking at speech to language transformations?

We've now tried to address this head on in the manuscript as below. Though in advance the answer is nuanced.

Given the availability of different speech models, one might wonder whether Whisper is currently the "best" model of brain activity. We consider that the answer to this question must in part be borne out experimentally through correlations between models and brain data. However, besides this, study aims may determine the appropriate model for use. For instance, Whisper's access to a preconfigured cross-lingual text vocabulary renders it an unsuitable model of childhood language acquisition when the word vocabulary also must be learnt (see also Millet et al. 2022). We chose to focus on Whisper because it provides an accurate end-to-end transformation of audio to text, and the potential to discover intermediary pre-lexical representations from audio data that have high utility for speech recognition. Thus, we considered it a good vehicle to address limitations of traditional hand-crafted and context-invariant categorical approaches that do not model how audio is categorized (Daube et al. 2019). Even though Whisper is trained with access to word units, we considered these to be more ecologically relevant units than phonemes - which humans don't explicitly need to know about to communicate.

However, the current analyses provide no clear answer on whether Whisper is the best EEG model right now. When compared to two self-supervised speech models (Wav2Vec2 and HuBERT) – which have no access to text in training but appear to induce lexical semantic representations in inner layers (Pasad et al. 2021, Vaidya et al. 2022), the top EEG prediction accuracies across layers were equivalent, albeit arising from the inner-layers of self-supervised models, rather than the last layer of Whisper's Encoder. In a variance partitioning analysis, Wav2Vec2 and Whisper overlapped in ~75% of EEG variance predicted. The ~15% extra variance contributed by Whisper might reflect Whisper's access to text in training. However, access to text in training appears not to benefit intracranial electrophysiological models of STG (Li et al. 2023). Relatedly, the inner layers of WavLM (Chen et al. 2022), a self-supervised extension of HuBERT, were found to mode fMRI data more accurately than Whisper's last layer, at least when accuracy was averaged across all brain voxels (Antonello et al. 2024). Thus, different speech modeling frameworks recover common representations that predict brain responses and provide convergent evidence that EEG reflects early contextualized linguistic transformation of speech, that are complemented by language models.

Minor:

1- Intro first paragraph: Add clarification on what "oracle" models are. Without this definition, the

sentence “This evidence has however been challenged…” comes out of nowhere and it’s difficult to understand what this is referencing (e.g. deleting this sentence entirely seems to have no effect as currently written, so if there is an important point here, the authors need to be more clear about it).

We’ve now deleted the explicit reference to oracle models – to make the article more self-contained and stream-lined (please see the earlier responses that paste in the amended part of the introduction).

2- In Figure 3, it would help to have the labels for which model was used beneath each of the “Unattended” brain panels.

We rejigged Fig 2 to hopefully make it clearer, as below.

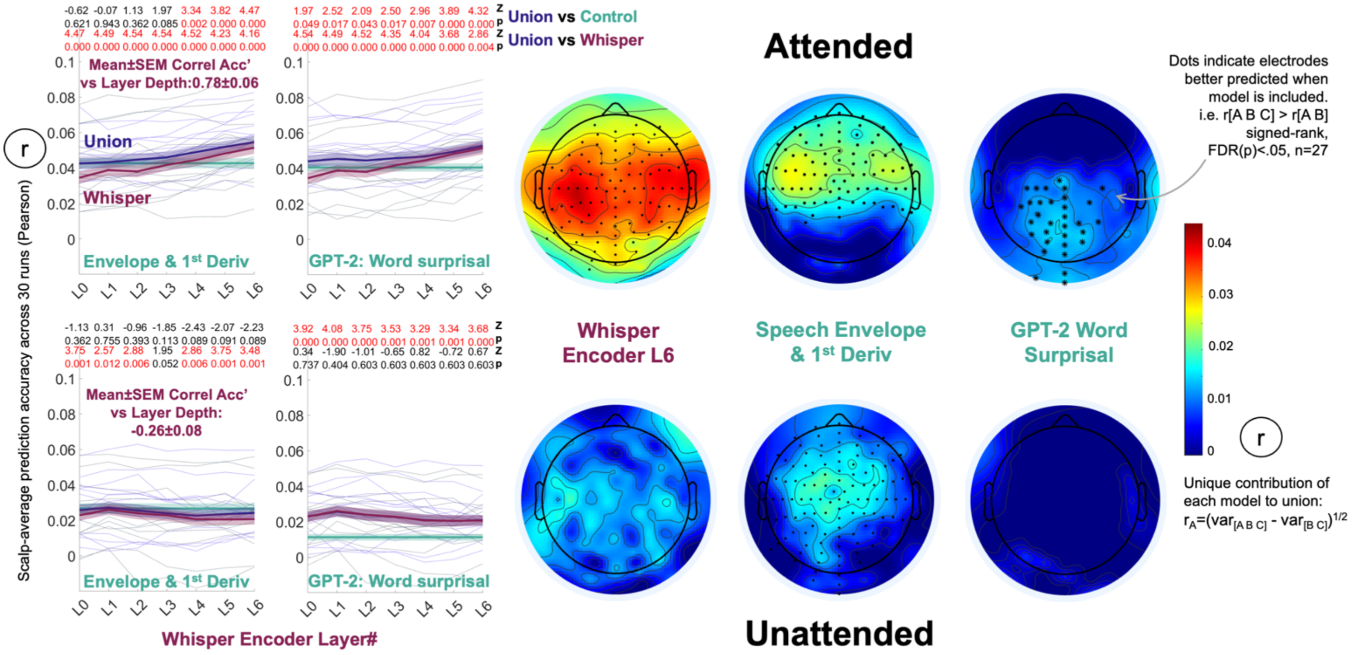

Thanks again for taking the time out to conduct this careful review !

Reviewer #4: In “Context and Attention Shape Electrophysiological Correlates of Speech-to-Language Transformation” Anderson et al. show that a deep learning system called Whisper can be used to generate representations that are particularly good at predicting EEG responses. Moreover, the predictive power increases (almost linearly) with the representations obtained from deeper and presumable more linguistic layers. In such, this study joins a growing body of work that are showing that large language models can be used as a tool to understand, or more candidly, assign functional representations to neurophysiological signals. The paper has many strengths. At the level of the presentation, I found the text to be easy read; it is concise but to the point and with very effective introductory and concluding paragraphs for each result. The figures are also well

designed in the sense of being highly informative. The methodology is also rigorous and, again, well explained. Finally, on a more substantive level, there is a significant effort put in the interpretation of the results. These additional analyses reveal, for example, the time scale of the context effects in speech related EEG responses (~10 s) or by manipulating the representations within words that some of the EEG prediction occurred at the sub-lexical level. I also appreciated the fact that Whisper was compared to the unsupervised deep nets, Wav2Vec2 and HuBERT. The comparison is not only useful to show that Whisper provides similar predictive power but because by contrasting representations in the different deep nets (or their training or architecture) one can again gain additional insights on what exactly is being represented in the neurophysiological signal. This manuscript will therefore serve as a nice example on how to effective leverage the power of deep NN in neurolinguistic research. I have a couple of major comments but since I have not analyzed EEG data myself (only fMRI and ECoG), I might be off track.

Thanks for the review and the encouraging remarks !

Major comments.
1. Spatial localization 1: One of the interesting results, which was not extensively developed, is that the GPT-2 word surprisal is not only able to yield additional predictive power (significant for lower layers) but that this additional power is found in a different spatial locations, in the centro-parietal electrodes. I am a bit puzzled by this result because I would expect a transformer based NN to be particularly good at capturing word surprisal as well. It would be interesting to explore this further, maybe by examining the correlations between word surprisal and the Whisper vectors?

Thanks for the thought here.

We've shied away from explicitly incorporating such an analysis in the manuscript, for two related reasons. First, modeling Whisper with lexical surprisal seems more relevant to deciphering Whisper than deciphering the EEG data (and based on the current analyses in Fig 2 we already know Whisper's Encoder doesn't capture components of lexical surprisal that are in the EEG data). And second, rather than looking at Whisper's Encoder for correlates of surprisal, Whisper's Decoder module (which we did not use in the current EEG analysis), is probably the natural place to start. This is because GPT-2 also is a Decoder network - that unlike Whisper lacks an Encoder (to contextualize speech for decoding). Thus, we foresaw a correlational analysis between Whisper Encoder vectors and surprisal (which would probably boil down to a time-lagged regression of whisper features on lexical surprisal) as heading off on a tangent.

However, we have now provided an overview of the workings of Whisper at an early stage in the Introduction (see below) – which hopefully sets up the reader with the idea that Whisper's Decoder is the (unused) piece of Whisper that resembles GPT-2. But different to GPT-2 which predicts next-words based on previous words alone (max 1024 tokens), Whisper's Decoder

predicts next-word identity with additional knowledge of the speech (up to 30s worth). Thus, Whisper's Decoder presumably produces qualitatively different surprisal estimates.

Operationally, Whisper turns continuous audio speech into categorical word units via a succession of intermediary transformations that take place within an "Encoder-Decoder" Transformer architecture (Vaswani et al. 2017). The Encoder module prepares input speech spectrograms for word decoding. This is achieved by re-representing each spectrogram timeframe as a "contextualized" weighted average of itself and all other time frames within a 30s window. The contextualization process is repeated across multiple intermediate layers, each feeding forward into the next. The output of the final layer, which is the output of the entire Encoder module is a time-series of contextualized speech vectors that are fed as one 30s chunk into the Decoder. The Decoder is also a multilayer feed-forward Transformer network, which then transcribes the encoded speech into a series of discrete word units in the same or a different language. This proceeds as an iterative process, with the decoder predicting the identity of the next word based on the encoded speech and any words it has previously decoded. Thus, the decoder closely resembles next-word-prediction language models such as GPT-2 (Radford et al. 2018), but with the additional access to contextually encoded speech.

Here, we model audiobook speech EEG recordings with Whisper's Encoder module, with an eye to identifying whether Whisper affords a predictive advantage over traditional acoustic measures, which we find, and then characterizing what underpins this advantage. We based our EEG analyses solely on Whisper's Encoder on the assumption that it plays the key role in transforming audio speech into a linguistic form to help the Decoder to predict word identity, otherwise the Encoder would be redundant.

2. Spatial localization 2: Given the results of word surprisal, it seems like it would also be interesting to generate electrode maps for all layers of Whisper. You did something like that for GPT-2 in supplemental Fig 5 but not for Whisper. I am guessing that you have tried that but that it was not particularly informative? Please comment.

Thanks for the suggestion, we have now added these maps in as a supplementary figure.

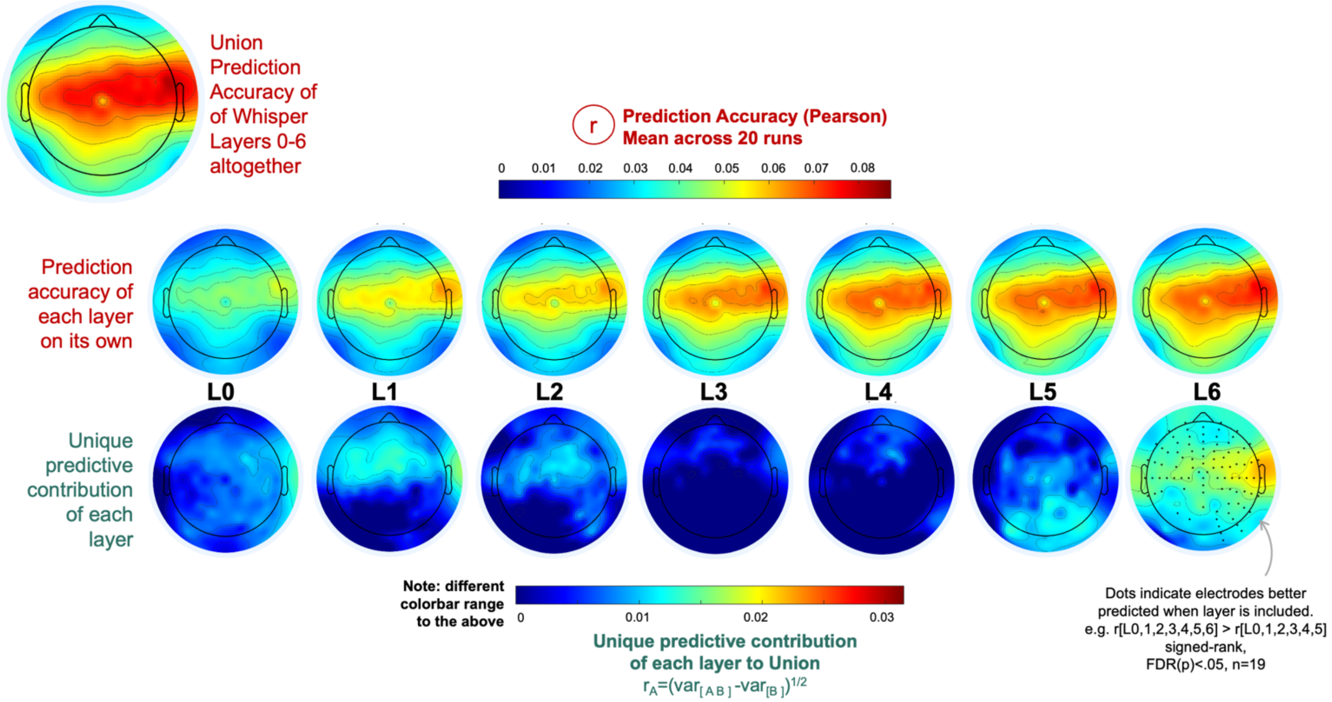

Supplementary Figure 6 (Figure 2 Companion). Whisper L6 encodes almost all information in earlier layers that is valuable for modeling EEG.

Top left: "Union Prediction Accuracy of Whisper Layers 0-6 altogether". Scalp map of electrode-wise EEG prediction accuracies derived from the Union of Whisper Layers L0-6. Mean±SEM Scalp-average prediction accuracy was 0.056+/-0.004.
Middle Row: "Prediction accuracy of each layer on its own". Scalp maps in the top row display electrode-wise prediction accuracies derived from Whisper Layers in isolation.

Bottom Row: "Unique predictive contribution of each layer". Scalp maps in the bottom row display the unique contribution each Whisper Layer made to EEG prediction, as evaluated with predicted variance partitioning. Whisper L6 was the only layer to independently contribute to prediction. L6 Mean±SEM Scalp-average prediction accuracy was 0.053+/-0.004. This was not significantly different from scalp-average prediction accuracy derived from the Union of L0-L6 (z=1.57, p=0.12, n=19, Signed-Rank test).

3. One analysis that it is missing and that I think could also be quite useful is to perform correlations between the vectors of the different layers of Whisper. Alternatively, one could also do a cumulative union and variance partitioning effect to further investigate what is being represented. Maybe doing this analysis and redoing a spatial analysis as suggested in point 2 would reveal interesting results?

Please see the above response, we combined both your suggestions 2 and 3 into Supp Fig 6. It turns out that most of the useful info is in Layer 6.

Minor comments.
1. I think that it is better practice to use R^2, the cross-validated coefficient of determination, instead of r to quantify prediction in statistical models.

Thanks for pointing this out – we see that using R^2 has benefits in taking into account how intercept and slope differ between observed and predicted, and current use of r^2 was made more out of habit than anything else. In the current case of normalized (z-scored) EEG data, the current r^2 approach does appear to be a close approximation of R^2. As an example, we replicated the Whisper Encoder L6 scalp map (left below) from Figure 2 with R^2 (right below). The numbers at the top of each plot indicate the range in values [min max], which are very similar. On these grounds we've stuck with the current set up here, but will make provision to change our habits in the future.

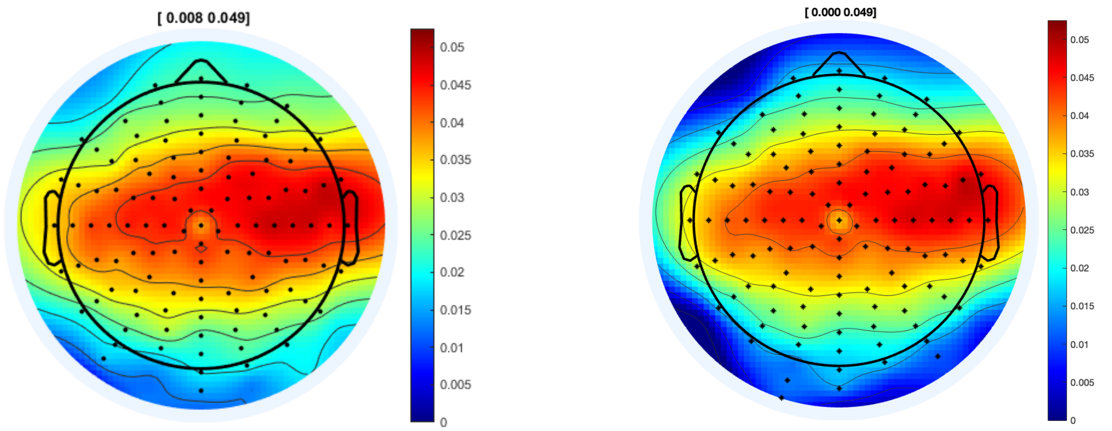

Also, if I understand correctly you are showing r values for a single trial (the 20th) and then average by permutation. I would make this clear in the figure legends as well and say it is a single trial r. This is to your advantage and is useful when comparing results from different studies.

This is now explicitly noted on the figure axes that correlation values correspond to averaged predictions across runs.

2. In Fig 2, the mean and sem for the control values appear to all be exactly the same for Env, Log-

Mel and GPT-2. There must be an error in the code that generated this figure (and hopefully not a cut and paste in illustrator ;-)

Yes, indeed they look similar, and they are more similar than we too had expected, but they are not precisely the same! All we can say is that this is the way the results turned out in the scalp averages with the current analysis framework. However, please note that different models differentially predict different electrodes. We've included a new supplementary figure with the individual results for readers who may ponder the same thing.

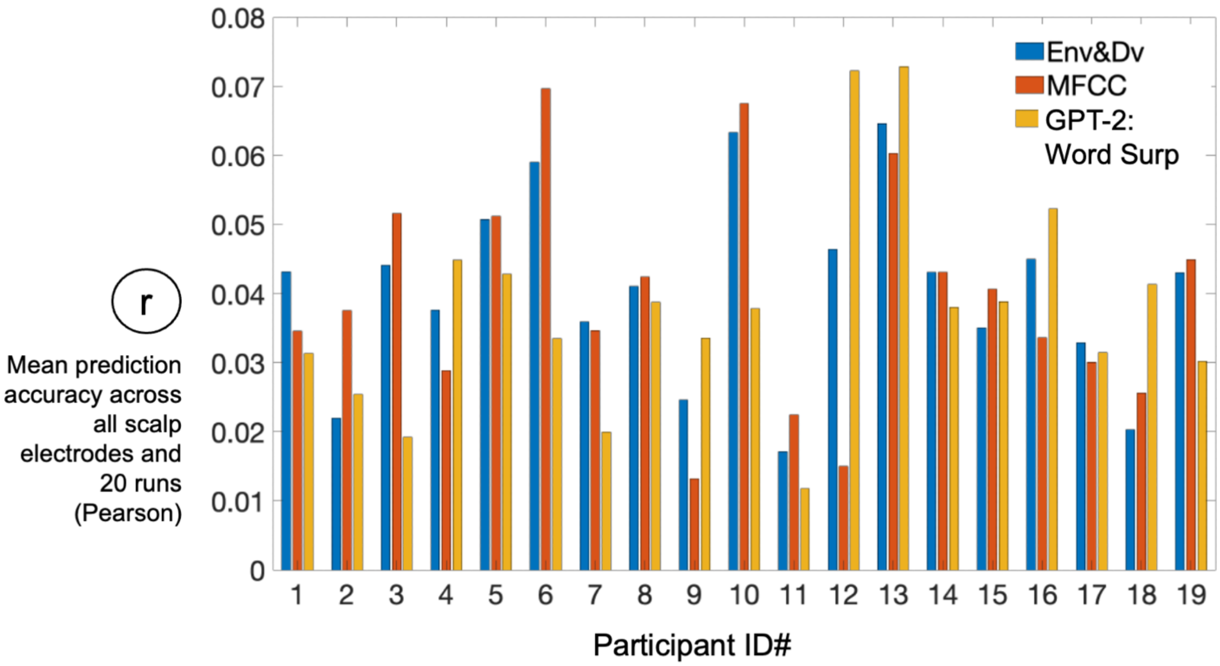

Supplementary Figure 5 (Figure 2 Companion). Individual prediction accuracies derived with Env&Dv, MFCC and GPT-2 Lexical Surprisal, to complement **Figure 2**, where Mean±SEM only are represented in green horizontal lines. The Mean±SEM prediction accuracies displayed in **Figure 2**, were: Env&Dv: 0.041±0.003, MFCC: 0.039±0.004, GPT-2 Word Surprisal: 0.038±0.004.

3. Some of the methods of Fig2 are repeated in the main text and could probably be eliminated in one or the other.

Thanks for the suggestion. I couldn't immediately figure out what best to delete, so kept it as it is.

4. Very minor suggestion: use A,B,C,D for figure labels instead of “mid-left”, “mid-right”.

We’ve done this for Figure 4, but did not for Figs 2 and 3, because they are already cluttered and to avoid confusion with the A,B,C in the variance partitions.

Frederic Theunissen.

Thank you again for the care, attention and time taken in writing this review !

Reviewer #5: The current article is describing an encoding analysis on EEG data of participants listening to natural speech. Specifically they use hidden states of a deep-learning speech recognition system as predictors of neural activity and compare it to a set of baseline models. I think the study is methodologically well executed, but the conclusions are still a bit unclear to me. I have two main points, one that is more severe and concerns what we learn from this study, and the second one more technical.

Thanks for the review and the positive comments. We’ve tried to clarify the motivation and conclusions of the study, as we outline below.

Phonemes as intermediary representations:

My major problem reading the current paper was to understand its contributions to understanding the “speech-to-language” transformation. The most prominent point that is mentioned e.g in the abstract is that “Contemporary models typically invoke speech categories (e.g. phonemes) as an intermediary representational stage between sounds and words. However, such models are typically hand-crafted and thus do not speak to the neural computations that putatively underpin categorization as phonemes/words.” or in the discussion: “a key limitation of previous work that has typically invoked hand-crafted categorical speech units such as phonemes as an intermediary phase, and thereby neglected modelling the mechanism that maps sounds to categorical units.”

We’ve now tried to clarify the nature of the article’s contribution in the Abstract

[snip] Contemporary models typically invoke context invariant speech categories (e.g. phonemes) as an intermediary representational stage between sounds and words. However, such models may not capture the complete picture because they do not model the brain mechanism that categorizes sounds and consequently may overlook associated neural representations. By providing end-to-end accounts of speech-to-text transformation, new deep-learning systems could enable more complete brain models. We model EEG recordings of audiobook comprehension with the deep-learning speech recognition system Whisper. We find that (1) Whisper provides a self-contained EEG model of an intermediary representational stage

that reflects elements of prelexical and lexical representation and prediction; (2) EEG modeling is more accurate when informed by 5-10s of speech context, which traditional context invariant categorical models do not encode; (3) Deep Whisper layers encoding linguistic structure were more accurate EEG models of selectively attended speech in two-speaker "cocktail party" listening conditions than early layers encoding acoustics. No such layer depth advantage was observed for unattended speech, consistent with the brain's more superficial level of linguistic processing.

...the Introduction

By enabling temporally precise estimates of brain activity, electrophysiological measures such as scalp EEG have provided evidence that the brain transforms natural continuous speech into words as a cascading process, with abstract categorical speech units such as phonemes or their articulatory features serving as intermediary pre-lexical representations (di Liberto et al. 2015, and more recently Gilles et al. 2023). To support this, researchers have typically revealed how EEG models of speech comprehension are improved when representing the speech stimulus as a time-series of categorical phoneme feature vectors in addition to the audio signal. However, the strength of this evidence has been critiqued (Daube et al. 2019) because: (1) Categorical speech models typically do not specify the computational mechanism that categorizes variable speech sounds, nor how sub-word representations are derived from audio data (because categories are manually configured by experimenters). They therefore may miss out on key transformational stages. (2) The phoneme predictive advantage may more parsimoniously be explained by phoneme timing (as can be approximated by taking the derivative of speech energy) rather than phoneme identity or articulatory structure.

...and the first paragraph of the Discussion.

The current study has revealed electrophysiological correlates of the linguistic transformation of heard speech using the end-to-end speech recognition model Whisper. This addresses a limitation of previous work that has typically relied upon hand-crafted context invariant categorical speech units such as phonemes to capture an intermediary phase between sound and words, and thereby neglected to model a mechanism that maps sounds to categories, and potentially also the representations invoked in this mapping. The current results suggest there is benefit to modeling EEG with a more complete model and suggest that this approach reveals correlates of a contextualized transformation that reflects both prelexical and lexical representation and predictive processing, and which cannot be comprehensively modeled by context invariant categorical approaches (by definition). To strengthen the case that the newly predicted EEG signal reflects a linguistic transformation as opposed to a brain-like filter of concurrent acoustic speech, the study further demonstrated that Whisper correlates were sensitive to listener attention. Specifically, correlates of Whisper's deeper more linguistic layers selectively diminished comparative to early layers when listeners ignored one speaker in favor of listening to another (for whom the correlates of deep layers were present). More generally, this study exemplifies how deep-learning models can help tackle unresolved questions in human speech comprehension, and in so doing predict neurophysiological data with minimal experimenter intervention.

Since phonemes are specifically mentioned as the 'old way of doing things' that this paper wants to improve on, I'm surprised that a phoneme encoding model is not at all considered as a comparison in the analyses. To me the nice thing about linguistic categories like phonemes or distinctive features (, place) is that they describe a possible intermediate representation that retains sufficient detail such that you can derive words from them (they distinguish minimal pairs). If the brain uses this representation, the neuroscientist can study smaller sub-computations, like how does the brain transform sound into phonemes instead of 'how does the brain transform sound into words'. I would like to understand what aspect of speech processing the new approach is capturing that previous approaches (e.g. phoneme encoding models) did not. Or whether they are capturing the same aspects but now do it in a more automatized way using a stimulus-computable model. Unfortunately this is not laid out clearly in the paper. Do the authors want to claim: the brain doesn't use phonemes, but a different intermediary representational format to map between sound and words, one that we cannot describe but is somewhere hidden in the connection weights of the model? Do they want to say that their model captures the 'mechanisms' that map sounds to phonemes, i.e. additional intermediary representations? Both claims would require some explicit comparison of Whisper-based to phoneme-based encoding models. Since the last author's group has used phoneme/feature models on this EEG dataset before (Di Liberto et al., 2015), it is surprising that this was not done.

If it turns out, say, that the Union of individual feature spaces (Envelope/Derivative + Phoneme labels and Word surprisal) predict EEG equally well predicted as the Whisper based encoding model, the authors could still say their model captures all of these features in a stimulus-computable way. I, personally, would think we learn more from considering individual 'hand-crafted' feature spaces. But it would make the scope of the study more clear.

Thanks for clarifying how the article was confusing.

I hope the comments pasted in above help clarify the contribution somewhat. We admittedly had found it tricky to tee up the contributions that the study makes without us having advance knowledge of the internal representations learnt by Whisper and have conducted a substantive rewrite to try to clarify much of this.

In response to the specific query about the phonemes – to clarify, our initial interest had been geared towards discovering the utility of end-to-end speech transformations, rather than refuting the explanatory value of phonemes. We also did not include the phoneme model in our initial analyses, in one part because the unique role it has to play in predicting the current EEG dataset comparative to audio measures has already been called into question (Daube et al. 2019). Therefore, in a sense this comparison is redundant. In another part, we present evidence that encoding context improves Whisper's EEG modelling accuracy, and this is something the context invariant categorical models don't have.

Nonetheless, we now include a comparative analysis in supplementary materials (below), which suggests that the phoneme model has little to add to the current EEG predictions beyond Whisper.

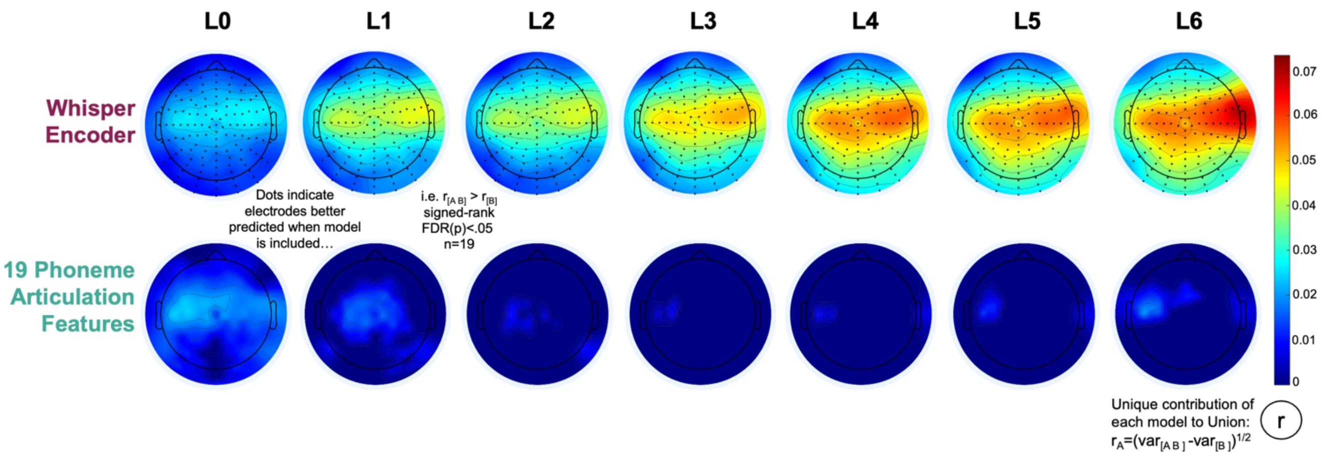

Supplementary Figure 7 (Figure 2 Companion). Whisper reflects almost all information in a phoneme articulation model that is useful for predicting EEG. The phoneme articulation model contains 19 binary articulatory features used in Di Liberto et al. (2015), which were resampled from the original 128Hz representation to 32Hz via nearest neighbor sampling. With this set up, predicted variance partitioning analyses found that each layer of Whisper could account for the EEG predictions made by the articulation model.

Computation of GPT2-based word surprisal:

As described in the Method section, GPT2 does not process words but so-called tokens. From what I understand this tokenization is done prior to GPT2's training in a data-driven manner, so does not correspond to linguistically meaningful units. The authors take a pragmatic step to aggregate surprisal values to match full words, which makes sense. However, the authors write that they average token surprisal values into a single word surprisal value. I think the correct thing to do would be to sum token surprisal values to get the word surprisal.

What we want is a word's (w) surprisal conditioned on the previous context (C), which is given by the negative log of it's contextual probability: -log p(w | C). Now if w is split into two tokens t_1 and t_2, we can get from GPT2 the contextual probability of the first token p(t_1 | C) and the contextual probability of the second token p(t_2 | C, t_1), which is conditioned on context C and the first token t_1. In order to compute p(w | C) we have to compute the joint probability of the two tokens by multiplying the token probabilities p(t_1 | C) * p(t_2 | C, t_1), using the chain rule of probability. So the surprisal should be either calculated as -log (p(t_1 | C) * p(t_2 | C, t_1)) or, equivalently, as -(log p(t_1 | C) + log p(t_2 | C, t_1)). In words, the surprisal of the full word in context should be the sum of the two tokens' surprisal.

Since I have not used GPT2 specifically, I can't judge how many words this applies to and how much this choice would effect the results. The averaging choice will systematically underestimate surprisal of the tokenized words: if many words are tokenized this could potentially underestimate the predictive value of GPT2-word surprisal in Figure 2. This should be checked and corrected. Of course, if there is a theoretical reason why the authors average rather than sum surprisal values, they should explain, but I can't see a good reason.

Thanks for spotting this. Sorry, it was a typo in the original manuscript! We had summed token estimates all along, rather than averaging. Sorry about the confusion. The manuscript is corrected as below.

As an addendum, to simplify the above explanation, we have implied that GPT-2 processes words. However more accurately, GPT-2 processes tokens which can either be words or sub-words (which can be useful to model new "out of dictionary" words). For instance, the word "skiff" is treated as two tokens: "sk" and "iff". In such a case GPT-2 would generate two token vectors for one word, and also two token-level surprisal estimates. In our analyses, the two token vectors were combined into a single word vector by pointwise summation – and the two token surprisal estimates were likewise summed to provide a single word surprisal estimate.

Minor points:

Description of Whisper. Although the Whisper architecture is described in detail in the Methods, some of the important aspects should be mentioned in the main text. Currently the Introduction and Results section describes it as a model that transforms speech into "language" (There are no line numbers in the text so it's difficult to refer to specific text, but this is repeated multiple times). This feels a bit imprecise: what is meant by language? Phonemes? Words? Meaning? It makes it hard to understand what the authors mean by the "later, more linguistic layers" without jumping to the Methods first. As I understand, Whisper is trained to generate word-level transcriptions using a separate decoder, even translating to a different language. This is important information, since it means that the later layers should represent very high-level aspects of language.

Thanks for the suggestion. That's fair. We've now revised the introduction to include the below to clarify this.

By modeling speech recognition end-to-end from audio to words, with human-like accuracy, recent deep artificial neural networks such as Whisper (Radford et al. 2022) present opportunities to alleviate concern (1) above and potentially provide a new window on speech comprehension in the brain. Critically, different to categorical speech models, intermediary representations within Whisper are a learned function of the audio spectrogram, that was optimized to reproduce speech transcriptions made by human annotators. Thus, Whisper might not only discover intermediary phoneme representations but also learn how to exploit phonetic and lexical context in service of speech recognition, which in turn might model new and/or known electrophysiological correlates of phonetic and lexical processing in natural speech

comprehension (e.g. di Liberto et al. 2015, Daube et al. 2019, Brodbeck et al. 2018, Broderick et al. 2018, Broderick et al. 2019, Heilbron et al. 2022). Indeed, recent empirical studies (Kloots and Zuidema 2024, Pouw et at. 2024) of the self-supervised speech model Wav2Vec2 (Baevski et al. 2020) have demonstrated sensitivity to phonological context and lexical knowledge, and more generally a range of different speech models have been observed to encode phonemes (Martin et al. 2023) and syntax and semantics to a degree (Pasad et al. 2024).

Operationally, Whisper turns continuous audio speech into categorical word units via a succession of intermediary transformations that take place within an "Encoder-Decoder" Transformer architecture (Vaswani et al. 2017). The Encoder module prepares input speech spectrograms for word decoding. This is achieved by re-representing each spectrogram timeframe as a "contextualized" weighted average of itself and all other time frames within a 30s window. The contextualization process is repeated across multiple intermediate layers, each feeding forward into the next. The output of the final layer, which is the output of the entire Encoder module is a time-series of contextualized speech vectors that are fed as one 30s chunk into the Decoder. The Decoder is also a multilayer feed-forward Transformer network, which then transcribes the encoded speech into a series of discrete word units in the same or a different language. This proceeds as an iterative process, with the decoder predicting the identity of the next word based on the encoded speech and any words it has previously decoded. Thus, the decoder closely resembles next-word-prediction language models such as GPT-2 (Radford et al. 2018), but with the additional access to contextually encoded speech.

Here, we model audiobook speech EEG recordings with Whisper's Encoder module, with an eye to identifying whether Whisper affords a predictive advantage over traditional acoustic measures, which we find, and then characterizing what underpins this advantage. We based our EEG analyses solely on Whisper's Encoder on the assumption that it plays the key role in transforming audio speech into a linguistic form to help the Decoder to predict word identity, otherwise the Encoder would be redundant. We further assumed that Encoder representations would become more linguistic with layer depth due to the feedforward architecture and contextualization process (see Methods for more details on these assumptions). However, the degree to which the learned linguistic forms reflect sub-words, words, or even semantics to successfully interface with the word decoder, and which of these features contribute to EEG models requires further investigation to estimate, which we undertook. To ease descriptions in the forthcoming text, we refer to the transformation performed by Whisper's Encoder as a speech-to-language transformation – with the proviso that language is ambiguously defined – and the conclusion we finally reach is that Whisper learns a mixture of sub-word, word structure, in part reflecting lexical predictions, and this helps to model EEG.

Thank you for the care and time taken in providing this review !

Review of "Context and Attention Shape Electrophysiological Correlates of Speech-to-Language Transformation"

In this study, the authors use sets of features drawn from the latent space of the WSPSR/Whisper network (Radford et al. 2022) to predict EEG data recorded while participants listened to audiobook passages in English. Using one dataset, the authors found that features drawn from the innermost layer of Whisper-base (L6) explained more of the neural response to speech than 1. the acoustic envelope

and its first derivative, 2. eighty mel-scaled spectrographic features, and 3. word surprisal values estimated with GPT-2. Additionally, using a dataset in which participants had been told to listen to only one of two simultaneously-presented audiobooks, the authors find that Whisper L6 features better explain the neural response to attended speech than unattended speech. For both datasets, the electrodes best explained by the Whisper feature sets were those typically associated with acoustic processing. Based on these findings, the authors argue that they find "new EEG correlates of speech-to-language transformation".

The application of Whisper-based feature sets to the decoding of scalp EEG data is novel. However, in the current manuscript, the authors' claims for the significance of their results are not sufficiently supported. I have three main critiques:

1. Key aspects of the research question are inadequately developed.
2. Some analytical choices are inadequately motivated.
3. Controls are generally inadequate for the claims made.

I outline each of these critiques in more detail below, alongside approaches towards strengthening future versions of the manuscript. However, given the substantiveness of the recommended revisions at this time, I do not think that this manuscript is ready to be considered for publication in PLOS Computational Biology.

Thanks for putting together this careful and attentive review.

1. Key aspects of the research question are inadequately developed.

Some terms that are central to the research question of this work are left poorly defined. Foremost of these, variations on the phrase "speech-to-language transformation" are used to describe both what the human brain does and what the Whisper encoding network does. Based on these usages, it is unclear what the authors think language is. Language is a topic of intense research, and there exist many well-described proposals for how it might work, including generative, categorial, and analogical proposals, among many others. Engaging with a recognizable theory of language I think will be crucial to better targeting the research question to strengthen this manuscript.

We have tried to more explicitly acknowledge that the representations of language in Whisper (which underpins the research question) lack any prior definition and require empirical investigation to pin down as below in the Introduction. NB New text cut directly from the manuscript is coloured, green, untouched manuscript text is in black font.

Here, we model audiobook speech EEG recordings with Whisper's Encoder module, with an eye to identifying whether Whisper affords a predictive advantage over traditional acoustic measures, which we find, and then characterizing what underpins this advantage. We based our EEG analyses solely on Whisper's Encoder on the assumption that it plays the key role in transforming audio speech into a linguistic form to help the Decoder to predict word identity, otherwise the Encoder would be redundant. We further assumed that Encoder representations would become more linguistic with layer depth due to the feedforward architecture and contextualization process (see Methods for more details on these assumptions). However, the degree to which the learned linguistic forms reflect sub-words, words, or even semantics to successfully interface with the word decoder, and which of these features contribute to EEG

models requires further investigation to estimate, which we undertook. To ease descriptions in the forthcoming text, we refer to the transformation performed by Whisper's Encoder as a speech-to-language transformation – with the proviso that language is ambiguously defined – and the conclusion we finally reach is that Whisper learns a mixture of sub-word, word structure, in part reflecting lexical predictions, and this helps to model EEG.

Similarly, one of the central claims of the manuscript is that it provides evidence of a novel EEG signature of language processing. Throughout the manuscript the authors also refer to an "EEG signature of Lexical Surprisal", "traditional N400 signatures of lexical processing", and "signatures of word processing". In these cases, it seems like the word *signature* is being used as a catch-all term for "brain measurement that correlates with X". In this way, the use of the term *signature* both cheapens the very precise behavior of the N400 response and oversells the (lack of) consensus in the field about neural correlates of lexical surprisal and lexical processing more generally. Moveover, in defaulting to a vague term like *signature*, the authors underdefine (and therefore likely undersell) their own contribution to the literature. I want more specific and precise descriptions of the significance of the results. It's easy to find a neural correlate of cognition; it's much harder to show that it appreciably improves the field's understanding of that cognitive domain. Therefore, the authors should focus more on convincing the reader that their neural correlate helps us understand something specific that is important about language.

Thanks for pointing this out. We have replaced "signature" with "correlates" throughout the manuscript.

We have also made extensive revisions to the manuscript to help clarify the contribution.

Abstract To transform continuous speech into words, the human brain must resolve variability across utterances in intonation, speech rate, volume, accents and so on. A promising approach to explaining this process has been to model electroencephalogram (EEG) recordings of brain responses to speech. Contemporary models typically invoke context invariant speech categories (e.g. phonemes) as an intermediary representational stage between sounds and words. However, such models may not capture the complete picture because they do not model the brain mechanism that categorizes sounds and consequently may overlook associated neural representations. By providing end-to-end accounts of speech-to-text transformation, new deep-learning systems could enable more complete brain models. We model EEG recordings of audiobook comprehension with the deep-learning speech recognition system Whisper. We find that (1) Whisper provides a self-contained EEG model of an intermediary representational stage that reflects elements of prelexical and lexical representation and prediction; (2) EEG modeling is more accurate when informed by 5-10s of speech context, which traditional context invariant categorical models do not encode; (3) Deep Whisper layers encoding linguistic structure were more accurate EEG models of selectively attended speech in two-speaker "cocktail party" listening conditions than early layers encoding acoustics. No such layer depth advantage was observed for unattended speech, consistent with the brain's more superficial level of linguistic processing.

Introduction First Paragraph The apparent ease with which the human brain transforms speech sounds into words belies the complexity of the task. This complexity is due in large part

to speech variability - each time a word is spoken, the sound is different. Speech variability is most striking in extreme cases such as when people have unfamiliar accents, shout, whisper or sing, but is always present to some degree, even when the same person repeats the same phrase (Liberman et al. 1967, Smith et al. 1995). How brains transform such variable speech sounds into language is a key unresolved question in cognitive neuroscience. By enabling temporally precise estimates of brain activity, electrophysiological measures such as scalp EEG have provided evidence that the brain transforms natural continuous speech into words as a cascading process, with abstract categorical speech units such as phonemes or their articulatory features serving as intermediary pre-lexical representations (di Liberto et al. 2015, and more recently Gilles et al. 2023). To support this, researchers have typically revealed how EEG models of speech comprehension are improved when representing the speech stimulus as a time-series of categorical phoneme feature vectors in addition to the audio signal. However, the strength of this evidence has been critiqued (Daube et al. 2019) because: (1) Categorical speech models typically do not specify the computational mechanism that categorizes variable speech sounds, nor how sub-word representations are derived from audio data (because categories are manually configured by experimenters). They therefore may miss out on key transformational stages. (2) The phoneme predictive advantage may more parsimoniously be explained by phoneme timing (as can be approximated by taking the derivative of speech energy) rather than phoneme identity or articulatory structure.

Discussion First Paragraph The current study has revealed electrophysiological correlates of the linguistic transformation of heard speech using the end-to-end speech recognition model Whisper. This addresses a limitation of previous work that has typically relied upon hand-crafted context invariant categorical speech units such as phonemes to capture an intermediary phase between sound and words, and thereby neglected to model a mechanism that maps sounds to categories, and potentially also the representations invoked in this mapping. The current results suggest there is benefit to modeling EEG with a more complete model and suggest that this approach reveals correlates of a contextualized transformation that reflects both prelexical and lexical representation and predictive processing, and which cannot be comprehensively modeled by context invariant categorical approaches (by definition). To strengthen the case that the newly predicted EEG signal reflects a linguistic transformation as opposed to a brain-like filter of concurrent acoustic speech, the study further demonstrated that Whisper correlates were sensitive to listener attention. Specifically, correlates of Whisper's deeper more linguistic layers selectively diminished comparative to early layers when listeners ignored one speaker in favor of listening to another (for whom the correlates of deep layers were present). More generally, this study exemplifies how deep-learning models can help tackle unresolved questions in human speech comprehension, and in so doing predict neurophysiological data with minimal experimenter intervention.

The use of imprecise language contributes to a research question that at times seems distracted and poorly motivated. While the introduction sets up a research goal of demonstrating the superiority of learned feature sets over a specific genre of handcrafted feature sets (those involving categorical speech sound labels), this is never tested.

Thanks for pointing out how this wasn't clear. We had not included the hand-crafted phoneme model in our initial analyses, because the unique role it has to play in predicting the current EEG

dataset comparative to audio measures has already been called into question (Daube et al. 2019), and the analysis may be redundant. We initially did not make that clear. For completeness we have run a comparative analysis with the phoneme model which is in supplementary materials. Whisper seems to account for all info in the phoneme articulation model that is useful for predicting EEG.

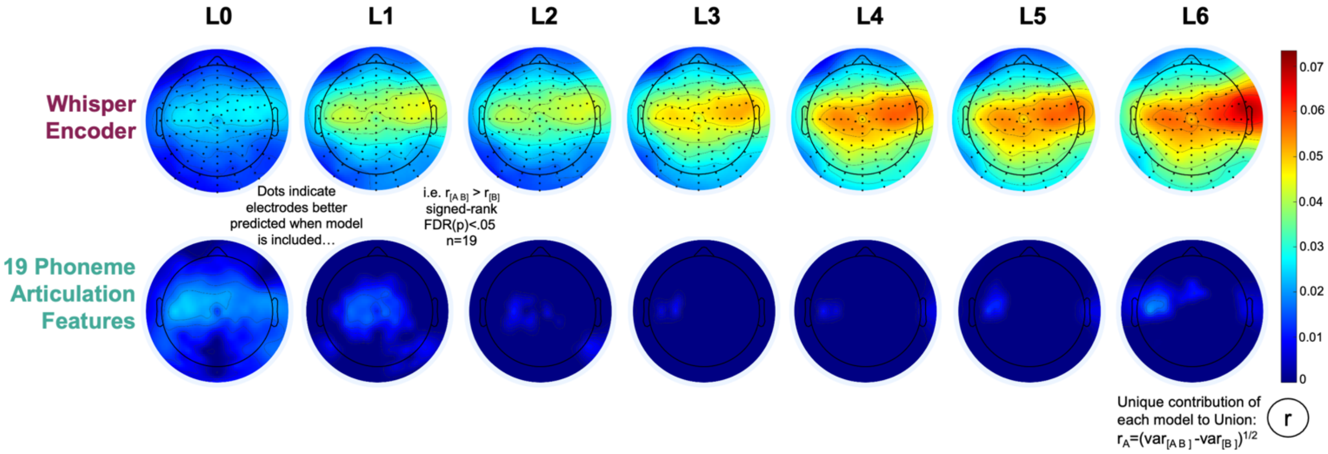

Supplementary Figure 7 (Figure 2 Companion). Whisper reflects almost all information in a phoneme articulation model that is useful for predicting EEG. The phoneme articulation model contains 19 binary articulatory features used in Di Liberto et al. (2015), which were resampled from the original 128Hz representation to 32Hz via nearest neighbor sampling. With this set up, predicted variance partitioning analyses found that each layer of Whisper could account for the EEG predictions made by the articulation model.

Since the introduction does not motivate predictions for the comparisons that are presented in the results section, predictions described in the results often seem ad hoc and poorly motivated. For example, on page 15 of the PDF the authors write, "Given the current evidence that EEG responses captured by Whisper reflect both lexical and sub-lexical structure we further examined how their timing related to responses to acoustic speech processing and language with the natural expectation that Whisper would be intermediary." Why this is a natural expectation is unexplained: If Whisper reflects both lexical and sub-lexical structure, why would its peak explanatory latency be expected to be intermediary and not double-humped?

Thanks for this interesting suggestion – we've outlined our rationale and this double humped possibility in the below.

Interpretation: Whisper best predicts EEG responses that are intermediary between speech acoustics and language

Given the current evidence that EEG responses captured by Whisper reflect lexical and sub-lexical structure we further examined how their timing related to acoustic speech processing and language with the expectation that Whisper would be either intermediary (reflecting a sub-lexical/lexical feature mixture) or would separably reflect both. To explore this, we ran a set of analyses where only a single time-lag of model features was used to predict EEG, rather than all time-lags at once as in our other analyses. Single

lags were within the range [0 to 750ms] in 1/32s steps. We reasoned that prediction accuracies derived from different lags would provide an estimate of the EEG response time-delay associated with each model. We were especially interested to see if such an analysis would show a single peak at intermediary lags (indicating an intermediary sub-lexical/lexical feature mixture) or whether it would show a double peak (suggesting separable indexing of sub-lexical and lexical features). Model-to-EEG mappings were fit on isolated models without variance partitioning to simplify analyses, and because stimulus features that are shared across models might be encoded at different stimulation latencies. In turn, this may exaggerate estimates of models' unique predictive contribution (NB the multi-lag regression analyses presented in the other results account for this). Therefore, for completeness we include a single-lag variance partitioning analysis in **Supplementary Figure 12**.

Related to these issues, the work engages inconsistently with appropriate literature. Three areas stand out.

1. Despite the fact that the study does not involve ERPs and Whisper features are not expected to contain information that the N400 would be sensitive to, the authors appeal to literature on the N400 throughout the paper. In one such instance on the middle of PDF page 6 the authors conflate the context sensitivity of the N400 with studies interested in phonological context (i.e., di Liberto et al. 2015 and Brodbeck et al. 2015), seemingly criticizing papers researching phonetics/phonology for not adequately integrating lexicosemantic context into their analyzes. This is a baffling argument.

We have removed this from the introduction – although it seems reasonable to us to entertain the idea that lexicosemantic content can guide phonological prediction. For instance, given a sentence beginning: "an apple grows on a", we consider it reasonable to expect the next sound will be 't' given the next word is likely to be 'tree'. We have added a couple of extra sentences into the Methods to clarify this.

In the case of modelling acoustic speech, context could be helpful to disambiguate noisy or mispronounced sounds, either within or across words. e.g. "television" might be inferred from the mispronunciation "televisiom" and in the case of the "the apple grew on the <noise>", the obscured word/sound is likely to be "tree". Interestingly recent studies of Wav2Vec2 (that unlike Whisper was trained without a language modelling objective) have revealed sensitivity to phonological context (Kloots MH, Zuidema 2024, Pouw et at. 2024) and lexical identity but not semantic context (Pouw et at. 2024).

2. The paper starts by describing the invariance problem, but only cites Smith (1995). This is a bit odd since discussion of the invariance problem goes back at the very least to the 1960s (see Liberman et al. 1967), and Smith (1995) is not a particularly seminal paper in that literature.

We have now cited Liberman et al. 1967.

3. More recent papers in the computational cognitive neuroscience of language tend to be cited as expected. However, in their results using the attention dataset, the authors write that "unlike EEG correlates of acoustic speech, the new EEG speech-to-language signature diminished when listeners ignored one speaker in favor of listening to another competing speaker". Here, the authors are contrasting their results with those of i.e., Mesgarani & Chang (2012), but seem to misstate either what Mesgarani & Chang show or what they themselves show. Notably,

Mesgarani & Chang show that unattended speech is less accurately reconstructed from neural data. Similarly, the current study also shows that Whisper features are less successful at reconstructing the neural response to unattended speech.

Thanks for pointing this out, we had never intended to appear to be refuting Mesgarani and Chang's work or indeed all the other studies. We have reworded appropriate sections of the manuscript.

In the Abstract.

Deep Whisper layers encoding linguistic structure were more accurate EEG models of selectively attended speech in two-speaker "cocktail party" listening conditions than early layers encoding acoustics. No such layer depth advantage was observed for unattended speech, consistent with the brain's more superficial level of linguistic processing.

In the Introduction.

We analyzed both audiobook comprehension EEG recordings made in: (1) single-speaker listening conditions, and (2) an experimental "cocktail-party" scenario where participants listened to two concurrent audiobooks but paid attention to only one (O'Sullivan et al. 2014). Extrapolating from Broderick et al.'s (2018) finding that correlates of lexical processing selectively reflect only the attended audiobook, we hypothesized the same would be true for deeper-more linguistic Whisper layers, whereas correlates of lower-level acoustics would remain for both attended and unattended speech, albeit to different degrees (Mesgarani and Chang 2012, Ding and Simon 2012, O'Sullivan et al. 2014, Fiedler et al. 2019).

In the Results

One effective way of modulating a listener's engagement to speech is via selective attention. In so-called cocktail-party scenarios it is widely appreciated that listeners can hone in on a single speaker whilst ignoring others (Mesgarani and Chang 2012, Ding and Simon 2012, O'Sullivan et al. 2014). Indeed, the electrophysiological bases of selective attention in multi-talker environments have been thoroughly investigated and there is general agreement that unattended speech is processed at a more superficial level than attended speech (Mesgarani and Chang 2012, Ding and Simon 2012, O'Sullivan et al. 2014, Broderick et al. 2018, Fiedler et al. 2019). For instance, listeners have low ability to accurately report on the unattended speech content, and N400-like lexical expectation responses disappear for unattended speech (Broderick et al. 2018) whilst traces of low-level acoustic speech processing remain, albeit at a diminished level (O'Sullivan et al. 2014). We therefore hypothesized that correlates of Whisper observed in **Figure 2** would be pronounced for the attended speaker in deep layers, but would dwindle or disappear for unattended speech, whereas acoustic correlates would remain to some degree. In particular, we hypothesized that the predictive advantage associated with Whisper layer-depth (the slope across layers in **Figure 2**) would flatten out for unattended speech.

2. Some analytical choices are inadequately motivated.

This section covers a handful of analytical choices that lack sufficient motivation in the text.

1. In Figure 1, the significance of the bottom row of plots is unclear. At the very least, the description of the plot axes should be clearer. I understand that the color in the bottom left of the matrix plot is intended to show that some attention weights are non-zero for words prior to the current word, indicating integration of previous information into current activations. Is the model weighting itself significant in some way? If so, on what basis are certain weightings significant? It may be helpful to see some kind of legend showing the range of weight values in the plot.

We considered taking this component of the Figure out, but decided against it, in that it gives a sense that the model is taking context into account in its computations, rather than just weighting neighboring speech on the diagonal. So, what you understood from viewing the matrices is essentially what we wished to convey. Thanks for pointing out that we'd omitted the range. We've added that into the caption. Up front we do not know which values in the matrices played a significant role in the current analyses.

2. At several points, models are claimed to yield "highly accurate predictions", but the maximum r value in the study appears to be 0.1, even for per-electrode (non-scalp average) measurements. These r values are on par with (if not a little lower than) values reported for analyses on this very same dataset in di Liberto et al. (2015). Di Liberto et al. use simpler, handcrafted feature sets. So, on what basis are the models in this study "highly accurate"? Even if this language is simply exaggeration, I think it's worth confronting di Liberto et al.'s results head on: do the Whisper features better explain the data at certain electrodes perhaps? or do they explain the same data as Di Liberto et al.'s handcrafted feature sets?

We removed references to "high accuracy prediction" throughout, also in light of another reviewers' comments. Please also note that Di Liberto et al's averages were not scalp averages (as reported in the current analyses) but averages of just the best electrodes and therefore likely to be inflated. As in our earlier response Whisper seems to capture the vast majority of EEG variance predicted by the phoneme model.

3. In Figure 2, the r values of the Env&Dv, Log-Mel Spectrogram, and GPT-2 word surprisal are practically identical. This is unexpected; why could this be? If I understand correctly, the Env&Dv feature set has two features per time bin, the Log-Mel Spectrogram feature set has 80 features per time bin, and the GPT-2 word surprisal model has one feature per time bin. It seems odd that 80 spectrographic features are not noticeably more informative of neural response than two acoustic correlates of loudness or one lexical statistical measure.

We were a little surprised too. However, this just turns out to be the way it came out for the scalp averages under the current analytic approach (but of course different models predict different scalp electrodes). We have included the individual-level scalp average results in Supplementary Materials.

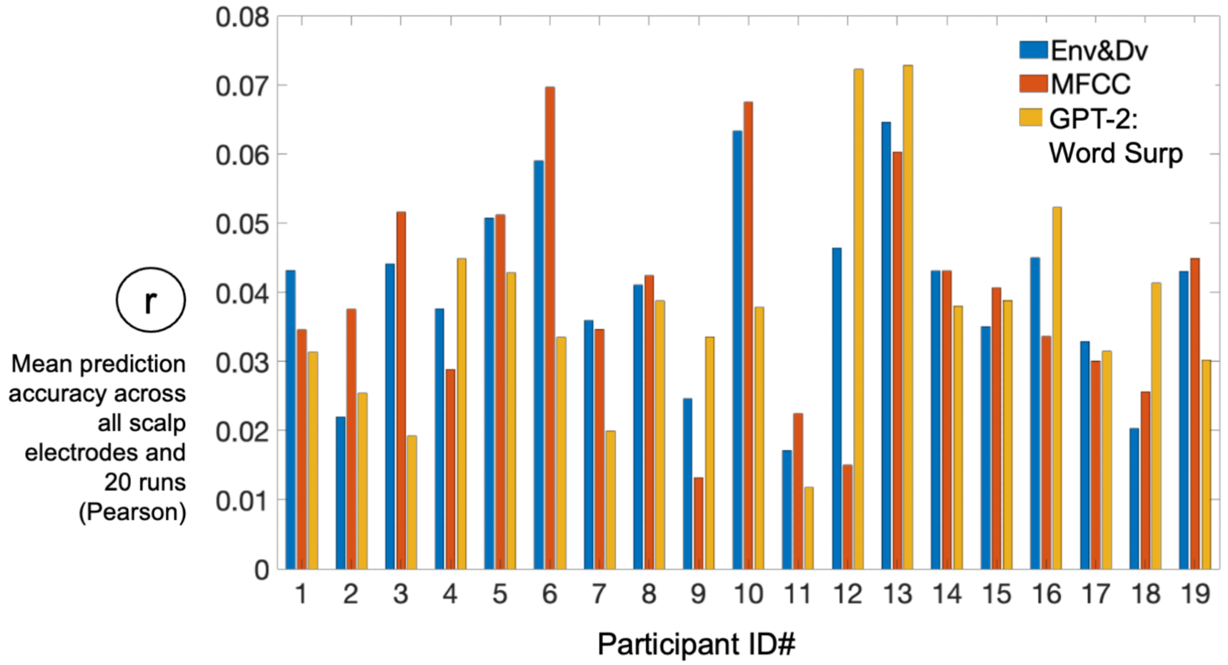

Supplementary Figure 5 (Figure 2 Companion). Individual prediction accuracies derived with Env&Dv, MFCC and GPT-2 Lexical Surprisal, to complement **Figure 2**, where Mean±SEM only are represented in green horizontal lines. The Mean±SEM prediction accuracies displayed in **Figure 2**, were: Env&Dv: 0.041±0.003, MFCC: 0.039±0.004, GPT-2 Word Surprisal: 0.038±0.004.

Overall for Figure 2 what I see is that the Left plot tells me that envelope and first derivative are features that Whisper does not extract by itself. The Middle plot tells me that Whisper already has log-mel spectrogram information (which is known from how Whisper is trained). The Right plot tells me that although longer range statistical/acoustic features of speech are extracted in deeper layers of Whisper, word surprisal itself is not something that Whisper extracts. Altogether, these insights characterize more about what information the Whisper feature set contains than how the brain processes language. This raises one of my biggest concerns about this study: the double black box problem. Throughout the paper, it seems like Whisper is mostly a fancy feature engineering technique, with the unfortunate downside that we know relatively little about what in those engineered features drives their modest ability to predict brain data. All we can say from this paper is that Whisper doesn't completely recapitulate Env&Dv information, word surprisal, or GPT-2 layer 16 information. Unfortunately this poses real challenges for the significance of the results to cognitive neuroscience: are the brain data being used as evidence about the structure of the Whisper network, or are the network features telling us something about the brain? As a potential PLOS Comp Bio paper, I would hope for the latter, but in this paper I mostly see the former. This is a hard problem in computational cognitive neuroscience right now, and it resists easy solutions. All I can recommend for now is that the revised manuscript will likely be stronger if it exploits something concretely known about the structure of Whisper and/or Whisper-derived features as a lever to explain brain data.

Whilst we agree that the current analysis tells us something about Whisper, we contend the comment above neglects the most salient finding that Whisper predicts information in EEG that these other approaches do not. We rewrote the abstract to seek to clarify this.

Abstract To transform continuous speech into words, the human brain must resolve variability across utterances in intonation, speech rate, volume, accents and so on. A promising approach to explaining this process has been to model electroencephalogram (EEG) recordings of brain responses to speech. Contemporary models typically invoke context invariant speech categories (e.g. phonemes) as an intermediary representational stage between sounds and words. However, such models may not capture the complete picture because they do not model the brain mechanism that categorizes sounds and consequently may overlook associated neural representations. By providing end-to-end accounts of speech-to-text transformation, new deep-learning systems could enable more complete brain models. We model EEG recordings of audiobook comprehension with the deep-learning speech recognition system Whisper. We find that (1) Whisper provides a self-contained EEG model of an intermediary representational stage that reflects elements of prelexical and lexical representation and prediction; (2) EEG modeling is more accurate when informed by 5-10s of speech context, which traditional context invariant categorical models do not encode; (3) Deep Whisper layers encoding linguistic structure were more accurate EEG models of selectively attended speech in two-speaker "cocktail party" listening conditions than early layers encoding acoustics. No such layer depth advantage was observed for unattended speech, consistent with the brain's more superficial level of linguistic processing.

3. Controls are generally inadequate for the claims made.

This section contains more targeted questions about the analysis and how particular analyses are interpreted.

1. What was done to control for the fact that most of the control models have fewer parameters than the Whisper model?

To attempt to circumvent overfitting, we ran regularized regression analyses in a cross-validation framework. Please note that the only models that complemented Whisper in EEG prediction had fewer features (Env&Dv and Lexical Surprisal). GPT2 L16 had the same number of features following PCA reduction (10) and MFCC had the most features of all (80). Additionally, intercomparisons between model layers were controlled in the sense that model layers all had the same number of features. Finally, within model comparisons were made across attended and attended speech conditions in the cocktail party analyses.

2. On PDF page 10, the authors write that when fitting models for EEG responses to unattended speech, "traces of low-level acoustic speech processing remain (Broderick et al. 2018), albeit with a reduced magnitude". Poorer fit of acoustic models on the brain response to unattended speech is also reported in Mesgarani & Chang (2012). The general consensus in the literature is that attention increases the fidelity of acoustic encodings of speech. That being said: Whisper is a transformer encoder that takes purely acoustic information as input and is trained to compress

that information in a way that can be most accurately used to mark word boundaries and map speech to text. Thus, the simplest explanation for the decrease in Whisper L6 performance when predicting the neural response to unattended speech is that Whisper provides a sophisticated compression of acoustic features, features which are less faithfully tracked by the brain when not attended to. In other words, Whisper is not sensitive to attention in the brain, but to acoustic features, the neural availability of which is modulated by attention.

Thanks for spotting that, we had not intended to suggest that Whisper is sensitive to human attention. We have revised much of the commentary surrounding the cocktail party analysis to emphasise that the attention effect is sensitive to whisper layer depth (please see earlier responses). More specifically, this is to say that the speech-to-language transformations that necessarily must happen for us to understand speech, do not happen to the same extent for unattended speech (because we don't understand it). And because Whisper is doing some speech-to-language transformations, its ability to model unattended speech does not improve across layers.

3. Why does shuffling or averaging the Whisper L6 features improve the fit for six of the participants in Figure 4?

We presume, because for those participants EEG recordings are capturing lexical but not sub-lexical representation. This could be due to differences in cortical folding between human participants, EEG recordings from different people likely contain different relative contributions from different (functionally specialized) cortical regions, meaning that the EEG of different people could reflect different speech and language representations to varying degrees. We have stated this in the text.

When Whisper L6 vectors were randomly shuffled within words, and this process was repeated 20 times, the unshuffled prediction accuracies (0.0534±0.0034, when averaging shuffles with participant) were found to be greatest in 13/19 participants. The cumulative binomial probability of achieving this outcome (p=1/20) in 13 or more participants is 2.5e-13. We presume that EEG recordings in the other 6 participants reflected correlates of lexical representation coded in Whisper. This could be due to differences in cortical folding between human participants, EEG recordings from different people likely contain different relative contributions from different (functionally specialized) cortical regions, meaning that the EEG of different people could reflect different speech and language representations to varying degrees.

4. Additionally for the shuffle manipulation depicted in Figure 4, I would expect shuffling vectors to decrease model performance generally, so I would like to see that it actually matters that the vectors are shuffled "within word" and not just any random comparably sized set of vectors getting shuffled.

We added in an extra analysis shuffling Whisper vectors across the entire timeline – to demonstrate that Whisper still adds predictive value when vectors are shuffled within words.

To establish the effects of disrupting within word structure, we lexicalized or shuffled Whisper L6 vectors within words, as described above. We then ran comparative cross-validation analyses, first predicting EEG data with the Union of [Whisper L6 Env&Dv Lexical Surprisal] and then

repeating analysis, but replacing Whisper L6 with either its shuffled or lexicalized counterpart. To provide confidence that L6 words with shuffled temporal structure still had some value for predicting EEG, we repeated analyses shuffling Whisper vectors across the entire timeline, which ablated Whisper's unique contribution to prediction altogether.

Consistent with the EEG data also reflecting sub-lexical structure, both experimental manipulations of Whisper damaged prediction accuracy for most participants (**Figure 4c**). Specifically, signed ranks comparisons of scalp-average prediction accuracies between Whisper L6 and lexicalized Whisper L6 revealed a significant drop in the latter (Mean±SEM=0.059±0.004 and 0.056±0.004 respectively, Z=2.86, p=0.0043, n=19, 2-tailed). When Whisper L6 vectors were randomly shuffled within words, and this process was repeated 20 times, the unshuffled prediction accuracies (0.0534±0.0034, when averaging shuffles with participant) were found to be greatest in 13/19 participants. The cumulative binomial probability of achieving this outcome (p=1/20) in 13 or more participants is 2.5e-13. We presume that EEG recordings in the other 6 participants reflected correlates of lexical representation coded in Whisper. This could be due to differences in cortical folding between human participants, EEG recordings from different people likely contain different relative contributions from different (functionally specialized) cortical regions, meaning that the EEG of different people could reflect different speech and language representations to varying degrees.

Ablating Whisper's contribution to prediction altogether by shuffling Whisper vectors across the entire timeline (without changing Env&Dv and Lexical Surprisal) produced Mean±SEM scalp-average prediction accuracies of 0.0380±0.0034 (when repeated 10 times with different random shuffles and averaging within each participant). This was significantly less accurate than predictions derived from within word shuffles (z=-3.8230, p= 1.32e-04, n=19, signed-rank) suggesting that Whisper still made a predictive contribution after within-word shuffling.

In sum, these analyses suggest that the current EEG correlates of speech-to-language transformation reflect a mixture of both lexical and sub-lexical structure in most participants.

5. As mentioned in Section 1 above, Figure 4 (right) is used as evidence that the Whisper feature set captures sub-lexical information intermediary to acoustics and lexical information. How does that follow from the data given? On PDF page 13, the authors write, "Consistent with EEG reflecting traces of lexical processing we found that late linguistic Whisper layers captured all variance predicted by GPT-2." This argument suggests that two feature sets accounting for the same *amount* of the variance are accounting for the same variance, which is not in general true.

Sorry, that was unclear. We have revised the Figure 4 caption to clarify that this was a variance partitioning analysis – so the two feature sets were indeed probably accounting for the same information in EEG (because adding GPT-2 on top of Whisper did not improve prediction).

Consistent with EEG reflecting traces of lexical processing we found that late linguistic Whisper layers captured all variance predicted by GPT-2 (and more), because the Union model (with GPT-2 and Whisper) was no more accurate than Whisper L5 or 6 alone. Differently earlier speech-like layers were complemented by GPT-2.

6. As mentioned in both Sections 1 and 2 above, throughout the analyses it's unclear what the counter-hypotheses would be for many analyses. Based on the introduction and an unspecified "sub-lexical" concept introduced in Figure 4, the authors seem to want to claim that the Whisper L6 features better explain brain data than transparently categorical models of speech sounds. Why isn't this tested head-to-head?

Please see our earlier responses, and the comparison to the phoneme model.

7. On PDF page 13, the authors write that "accuracy was greatest at 10s, suggesting that intermediate contexts – which could support extraction of semantics and syntax– are valuable". Given the fact that Whisper is trained on exclusively acoustic information, it's unreasonable to jump straight to the idea that 10s latencies support semantic or syntactic information. It's far more likely that the efficacy of 10s contexts is related to cross-speaker normalization, F0 normalization across utterances within speaker, accent stabilization, or consistency of acoustic cues to word boundaries. If the authors would like to appeal to syntax or semantics in this manuscript, they should offer more evidence for its relevance.

Thanks for pointing that out, we agree and have revised the section to explicitly acknowledge this suggestion.

Also please note that in preparing the analysis code for upload to an open science forum, we spotted that the Whisper context window length analysis was run fitting a 0-600ms lag temporal response function, which is inconsistent with the remainder of results which are 0-750ms. We now present 0-750ms for consistency. There are minor differences in results (the 600ms TRF is a tiny bit more accurate for 10s context (r=0.57 compared to 0.56) and the 30s context window is no longer significantly less accurate than 10s context.

The section now reads as below:

Interpretation: EEG Preferentially Reflects the Encoding of 5-10s Speech Contexts

Because long multi-word speech contexts would seem to be necessary for Whisper to have captured EEG correlates lexical prediction above, we examined how valuable Whisper's 30s context was for modeling EEG data. To this end we generated Whisper L6 vectors using sliding context windows that we constrained to different durations [.5s 1s 5s 10s 20s 30s] to restrict Whisper's access to linguistic context. As is illustrated in **Figure 4b**, the strongest prediction accuracies were observed for 10s of context, and these accuracies were significantly greater than all other context window sizes shorter than 5s. However, although significant, the gain in prediction accuracy between 0.5s (Mean r=0.051) and 10s (Mean r=0.056) was modest, equating to 22% extra variance predicted (r^2). A subsequent exploratory analysis of individual electrodes' sensitivity to different context durations (**Supplementary Figure 11)** revealed that all electrodes were preferentially predicted with 5s or more context, and some posterior scalp electrodes were sensitive to lengthier Whisper contexts of 30s.

Critically, these results provide evidence that EEG signals are sensitive to multi-word speech contexts cannot be modeled by traditional context-invariant categorical approaches. However future work will be necessary to pin down precisely what contextual information was critical. Although speech models appear to encode elements of semantics and syntax (Pasad et al. 2024) that could support lexical and phonological predictions, it is also possible that the contextual advantage draws from cross-speaker normalization, F0 normalization across utterances within speaker, accent stabilization, or consistency of acoustic cues to word boundaries.

Miscellaneous

- PDF Page 6 middle of Paragraph 1: The author is Gillis, not Gillest. (It's correct in the bibliography)

Thanks for spotting – we've fixed it.

- What is the dotted black line in figure 4 Mid-left? Just the value for the 0.5s sliding window? I think this should be mentioned explicitly somewhere.

Yes, precisely that, we've now mentioned this in the caption, as below.

The dashed horizontal line reflects mean prediction accuracy with a 0.5s context.

- Also for Figure 4 (mid-left), in the figure it looks like 5s, 10s, and 20s lags are not significantly different from one another, but on PDF page 14 it says that only 10s and 20s were not significantly different from one another. What is correct?

Good catch, thanks for spotting this. Please also see our earlier response for the revisions made to this section to correct an accidental mis-parameterization of our initial analysis (the analysis is now run with a 0-750ms rather than the original results which were accidentally derived from a 0-600ms TRF). The upshot is that there is no statistically significant difference between 5 and 10s. We have revised the manuscript accordingly (please see the revised "**Interpretation: EEG Preferentially Reflects the Encoding of 5-10s Speech Contexts"** section in the above response)

In the abstract:

EEG modeling is more accurate when informed by 5-10s of speech context.

Fig 4 caption:

Mid-left: To examine whether Whisper's accurate EEG predictions were driven by contextualized representation, Whisper's context window size was constrained to different durations [0.5s, 1s, 5s, 10s, 20s, 30s]. Accuracy was greatest at 5-10s, suggesting that intermediate contexts spanning multiple words were beneficial. Corresponding signed ranks test Z and FDR corrected p-values are displayed on the plot. The dashed horizontal line reflects mean prediction accuracy with a 0.5s context.

● PDF Page 10: “listeners can home in on a single speaker” It should be ‘hone.’

Oops – corrected.

● In general, all figures would be improved with lettered subfigure labels (i.e., a, b, c)

Thanks. We did this for Fig 4. We kept Fig 2 and 3 as is, due to clutter and to avoid confusion with the variance partition description which is also described in terms of a,b,c,d.

● What do attended and unattended look like per audiobook condition?

We’ve added in supplementary figures with results specific to the two audiobooks.

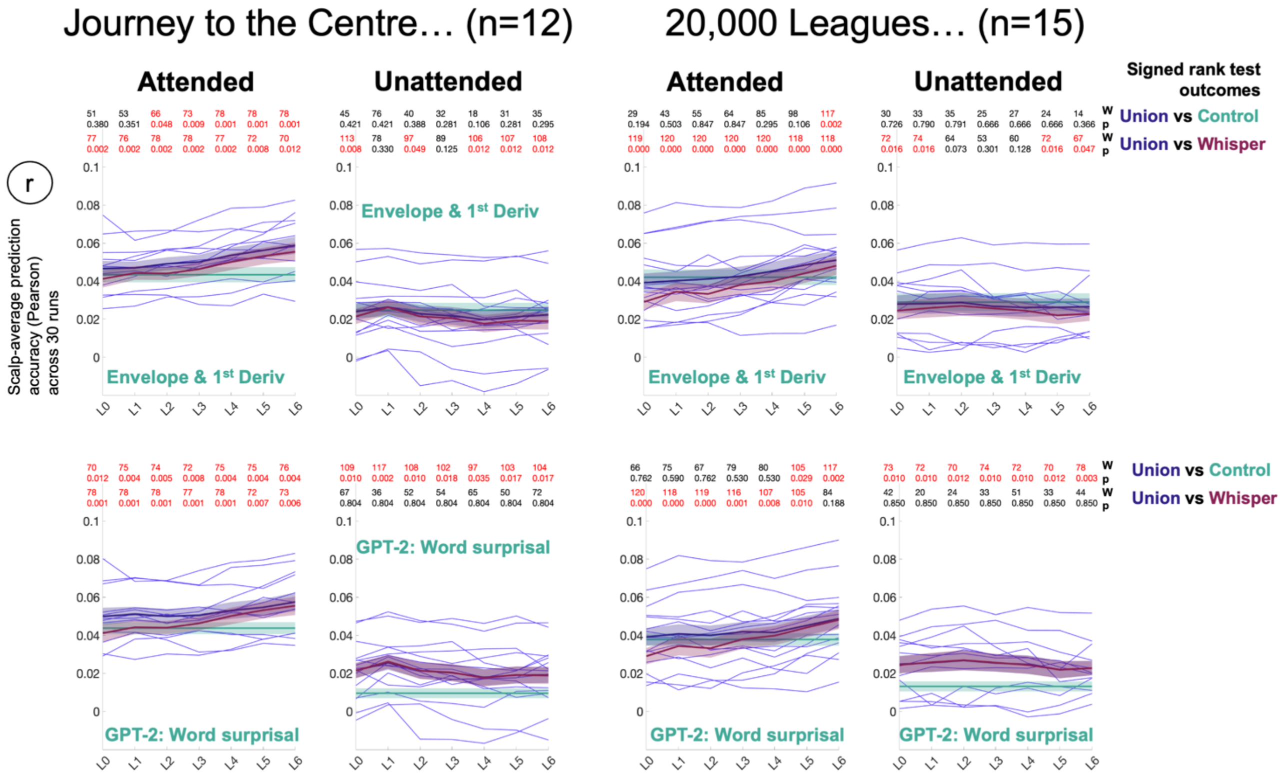

Supplementary Figure 8 (Figure 3 Companion). EEG correlates of selectively attended and unattended speech in two concurrent speaker (audiobook) “cocktail-party” conditions, splitting up **Figure 3 Left** by story (Journey to the Centre of the Earth and 20,000 Leagues under the Sea).

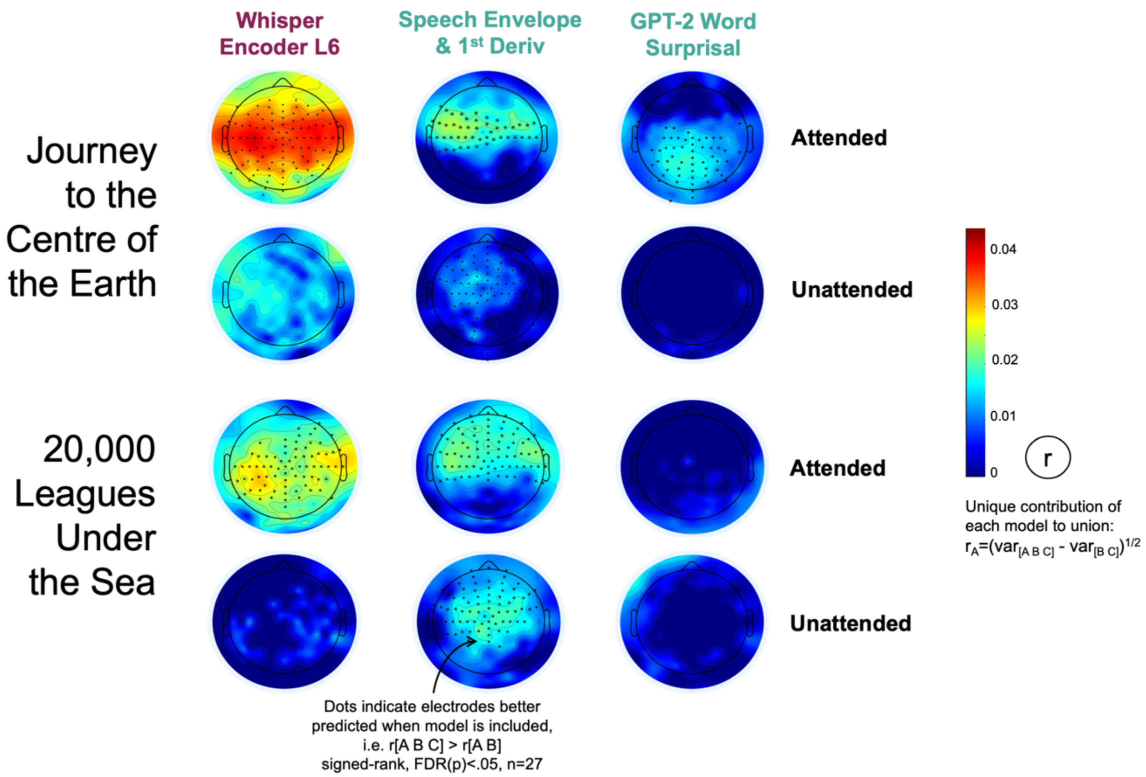

Supplementary Figure 9 (Figure 3 Companion). EEG correlates of selectively attended and unattended speech in two concurrent speaker (audiobook) “cocktail-party” conditions, splitting up **Figure 3 Right** by story (Journey to the Centre of the Earth and 20,000 Leagues under the Sea).

- Are signed ranks tests between Control and Whisper models reported somewhere? Did I miss them?

These are the row of numbers at the top of the figures, we have sought to emphasise this in the text and figure captions.

- PDF Page 15, Figure 5 Caption: “Wav2Vec2 and HuBERT both yielded highly accurate predictions but unlike Whisper, the inner layers were accurate.” Doesn’t Figure 5 show that inner layers of Wav2Vec2 and HuBERT are less accurate than Whisper?

Apologies for the confusion, the caption may have been misleading. We have clarified it to make the point that rather than the last layer, L7 and L9 were most accurate for Wav2Vec2 and HuBERT. NB: Wav2Vec L7 at least is statistically speaking no less accurate than Whisper L6.

The revised caption reads as:

Figure 5. To explore how Whisper's accurate EEG predictions compared to self-supervised speech models trained without direct access to language (on identifying masked speech sounds) we also repeated analyses with Wav2Vec2 and HuBERT. To enable cross-referencing to comparative fMRI studies (Millet et al. 2022, Vaidya et al. 2022), we performed this comparison on models comprising 12 layers. Wav2Vec2 and HuBERT both yielded highly accurate predictions but unlike Whisper, the inner layers (L7 and L9 respectively) rather than the last layer were most accurate. Further tests reported in the main text, suggest that Whisper predicted some different components of EEG signal to Wav2Vec2.

● PDF Page 15: "Please note also, that GPT-2 and probably Whisper (as above) are anticipatory, and may capture brain responses at short-latency." What is meant by this?

Thanks for pointing this out. We were alluding to GPT-2 making predictions, but it is not an essential sentence, so we deleted it for brevity.

● I personally wouldn't use PCA to compress the Whisper L6 feature space because it's a pretty crude and lossy compression technique. It's likely that the advantage of the PCA dimensions is maxing out at 10 just because the compression is poor quality. Have you tried training an autoencoder to reduce the dimensionality of the Whisper feature space?

Thanks for the suggestion. You may well be right, however we opted for using PCA for simplicity, especially to avoid running a separate cross-validated analysis on Whisper alone to fit the autoencoder.

● Supplementary Figure 5, top right: I don't think this style plot is best for a categorical measure. At the very least, the legend should be categorical.

Thanks, that's fair – to be precise, we've annotated the electrodes with the most accurate layer. We've kept the colourmap to give an instant impression of the patterns, even though it is admittedly imprecise. Please see the below cut out from the new Supplementary Figure.

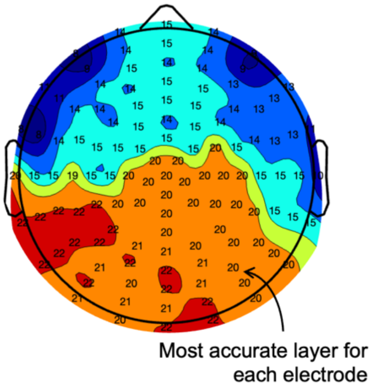

Thanks again for the time and careful attention put into this review !
