# Supplementary material for: Deep-learning models reveal how context and listener attention shape electrophysiological correlates of speech-to-language transformation
Source: PLoS Comput Biol. 2024 Nov 11;20(11):e1012537. doi: 10.1371/journal.pcbi.1012537 (PMC11581396; doi:10.1371/journal.pcbi.1012537)
Supplement: Attachment — Submitted filename: plos_compbio_nov_2023_v2-1.pdf [file pcbi.1012537.s004.pdf]

Review of “Deep-Learning Models Reveal Context and Listener Attention Shape Electrophysiological Correlates of Speech-to-Language Transformation”

Thank you to the authors for your exhaustive responses. They have made it much clearer where the paper is strong and where issues still remain. The paper continues to be strong in its detailed analyses, information-rich figures, and discussion of temporal dynamics of the neural response to speech.

I have two main remaining issues. As for the first issue, I think the contribution of the work to language research remains unclear, but I also recognize that I expect quite a bit of conservativeness and specificity in argumentation in language research, and that may just be on me. On those grounds alone, I could see the paper being accepted with maybe one last attempt to engage more carefully / precisely with language research.

However, the second issue is a bit more serious in my mind, because it’s not just a matter of rhetoric maybe, but of how the results presented here square with those reported previously on the same dataset (DiLiberto et al. 2015). I explain what I mean more below, but on those grounds, I still do not think this paper is ready for publication in PLOS Computational Biology.

1. Motivation and Engagement with Theoretical Literature

I’m not really satisfied with the responses to me and other reviewers who asked that the advantages and disadvantages of categorical phonemic feature sets and Whisper feature sets be better described. In particular, the revised text doesn’t really accurately convey what phonemes “are for” in the cognitive science and psycholinguistic literatures where they’re used. In engineering fields, they may just be seen as unlearned labels imposed by annotators, but in the study of language, they have real theoretical import, and do in fact imply a model of the relationship between acoustics and meaning-free sublexical components of speech (though the model specifics may vary from paper to paper).

Nevertheless, no language researcher expects that phoneme labels ought to explain the entirety of the neural response to speech, and yet that seems to be the position set up as an argumentative foil in the revised text. For example, in the abstract:

> Contemporary models typically invoke context invariant speech categories (e.g. phonemes) as an intermediary representational stage between sounds and words. ***However, such models may not capture the complete picture*** because they do not model the brain mechanism that categorizes sounds and consequently may overlook associated neural representations.

In the introduction:

> …[R]esearchers have typically revealed how EEG models of speech comprehension are improved when representing the speech stimulus as a time-series of categorical phoneme feature vectors in addition to the audio signal. However, the strength of this evidence has been critiqued (Daube et al. 2019) because: (1) Categorical speech models typically do not specify the computational mechanism that categorizes variable speech sounds, nor how sub-word representations are derived from audio data (because
> categories are manually configured by experimenters). ***They therefore may miss out on key transformational stages.***

And in the discussion:

> The current study has revealed electrophysiological correlates of the linguistic transformation of heard speech using the end-to-end speech recognition model Whisper. This addresses a limitation of previous work that has typically relied upon hand-crafted context invariant categorical speech units such as phonemes to capture an intermediary phase between sound and words, and thereby neglected to model a mechanism that maps sounds to categories, and potentially also the representations invoked in this mapping. ***The current results suggest there is benefit to modeling EEG with a more complete model*** and suggest that this approach reveals correlates of a contextualized transformation that ***reflects both prelexical and lexical representation and predictive processing, and which cannot be comprehensively modeled by context invariant categorical approaches (by definition).***

I'm pretty sure I'm not misrepresenting the intention of these lines here, since a response to Reviewer #3 also states that "the argument we had intended to convey was [that] modeling phonemes doesn't provide the complete picture." No language researcher thinks that modeling phonemes *does* provide the complete picture, so I don't think this is a convincing framing for the paper's contribution.

I think the bigger issue here comes back to a lack of engagement with a recognizable theory of language or language in the brain, which was the first point I made in my previous review. However, based on the response to that request, maybe it wasn't clear what I was asking for. Obviously, "the representations of language in Whisper…lack any prior definition and require empirical investigation to pin down"; that's their biggest weakness for the paper. It's still important to contextualize what the results mean for language, whether that's engaging with something like Hickok and Peoppel (2007) or Elan Dresher's chapter on the phoneme in the 2011 *Blackwell Companion to Phonology*. I don't think what's included in the revised manuscript is sufficient.

2. Replicate DiLiberto et al. (2015)

Thank you for adding a figure on the performance of categorical features relative to Whisper features to the text. I do think it's a bit odd that it's in the Supplementary Materials though. I

understand you weren't convinced it was necessary, but since the abstract, introduction, and discussion of the revised paper all continue to focus on the advantages of Whisper over categorical models, it seems a bit strange that the only figure addressing categorical models is in the Supplementary Materials.

That choice aside, it's not clear to me from Supplementary Figure 7 whether you were able to replicate DiLiberto et al.'s (2015) results. This for me is really important, especially considering the inability of the 80-feature spectrographic model to outperform the Env&Dv model. It got me thinking more generally about differences between your pipeline and DiLiberto et al.'s, and I realized that your choice of sampling rate may be adversely affecting the performance of your more time-sensitive feature sets (i.e., spectrographic features and phones).

In particular, it looks like work in this area that uses higher sample rates (>100Hz) shows more sensitivity to these feature types generally (e.g., DiLiberto et al. 2015, Gwilliams et al. 2022, Tezcan et al. 2023, Mai et al. 2024), compared to those using lower sample rates (e.g., this manuscript, Daube et al. 2019). This shouldn't be surprising considering that some phonemes are as little as 10ms in duration, and many of the acoustic correlates of phoneme identity (e.g., formant transitions) are similarly brief. With a sampling resolution of 32Hz, your time bins are over 30ms, which means that they don't include information that we know is important for speech processing.

What I would say is: try to replicate the content of DiLiberto et al.'s Figure 2A,B with your signal and features at 32Hz and 128Hz. If the results are qualitatively similar, then report that in the Supplementary Materials, and put somewhere in the text that you've validated that your pipeline parameters are reasonable for the features and data you're using. However, if DiLiberto et al.'s results can't be qualitatively validated at 32Hz, then you'll need to redo all analyses using an appropriate sampling rate.

Regardless of the outcome of the replication, there should be text in the discussion section addressing how the results of this work compare/contrast to DiLiberto et al.'s and the implications of that.
