# Supplementary material for: Deep-learning models reveal how context and listener attention shape electrophysiological correlates of speech-to-language transformation
Source: PLoS Comput Biol. 2024 Nov 11;20(11):e1012537. doi: 10.1371/journal.pcbi.1012537 (PMC11581396; doi:10.1371/journal.pcbi.1012537)
Supplement: Attachment — Submitted filename: response_to_reviews_2.docx [file pcbi.1012537.s005.docx]

Dear Dr Marinazzo,

Thank you very much for overseeing the review of our revised manuscript.

We have revised the manuscript according to the final reviewer’s comments and also conducted the extra analysis requested. The colourmap in the figures is now also changed throughout.

We again thank reviewers for their time and believe the manuscript to be substantially improved in response to their comments.

Please find below an itemised list of our revisions.

Thanks again for your interest in this manuscript, and we hope the revisions will be satisfactory.

Yours sincerely,

Andrew Anderson, Chris Davis, Ed Lalor

**Response below:**

**1. Motivation and Engagement with Theoretical Literature**

I’m not really satisfied with the responses to me and other reviewers who asked that the advantages and disadvantages of categorical phonemic feature sets and Whisper feature sets be better described. In particular, the revised text doesn’t really accurately convey what phonemes “are for” in the cognitive science and psycholinguistic literatures where they’re used. In engineering fields, they may just be seen as unlearned labels imposed by annotators, but in the study of language, they have real theoretical import, and do in fact imply a model of the relationship between acoustics and meaning-free sublexical components of speech (though the model specifics may vary from paper to paper).

Nevertheless, no language researcher expects that phoneme labels ought to explain the entirety of the neural response to speech, and yet that seems to be the position set up as an argumentative foil in the revised text. For example, in the abstract:

Contemporary models typically invoke context invariant speech categories (e.g. phonemes) as an intermediary representational stage between sounds and words. *However, such models may not capture the complete picture* because they do not model the brain mechanism that categorizes sounds and consequently may overlook associated neural representations.

In the introduction:

...[R]esearchers have typically revealed how EEG models of speech comprehension are improved when representing the speech stimulus as a time-series of categorical phoneme feature vectors in addition to the audio signal. However, the strength of this evidence has been critiqued (Daube et al. 2019) because: (1) Categorical speech models typically do not specify the computational mechanism that categorizes variable speech sounds, nor how sub-word representations are derived from audio data (because categories are manually configured by experimenters). ***They therefore may miss out on key transformational stages.***

And in the discussion:

The current study has revealed electrophysiological correlates of the linguistic transformation of heard speech using the end-to-end speech recognition model Whisper. This addresses a limitation of previous work that has typically relied upon hand-crafted context invariant categorical speech units such as phonemes to capture an intermediary phase between sound and words, and thereby neglected to model a mechanism that maps sounds to categories, and potentially also the representations invoked in this mapping. ***The current results suggest there is benefit to modeling EEG with a more complete model* and suggest that this approach reveals correlates of a contextualized transformation that *reflects both prelexical and lexical representation and predictive processing, and which cannot be comprehensively modeled by context invariant categorical approaches (by definition).***

I’m pretty sure I’m not misrepresenting the intention of these lines here, since a response to Reviewer #3 also states that “the argument we had intended to convey was [that] modeling phonemes doesn’t provide the complete picture.” No language researcher thinks that modeling phonemes *does* provide the complete picture, so I don’t think this is a convincing framing for the paper’s contribution.

I think the bigger issue here comes back to a lack of engagement with a recognizable theory of language or language in the brain, which was the first point I made in my previous review. However, based on the response to that request, maybe it wasn’t clear what I was asking for. Obviously, “the representations of language in Whisper...lack any prior definition and require empirical investigation to pin down”; that’s their biggest weakness for the paper. It’s still important to contextualize what the results mean for language, whether that’s engaging with something like Hickok and Peoppel (2007) or Elan Dresher’s chapter on the phoneme in the 2011 *Blackwell Companion to Phonology*. I don’t think what’s included in the revised manuscript is sufficient.

Sorry for the confusion here, we had not been seeking to give the impression that anyone thinks phonemes are a complete model of neural speech responses, and we may have unintentionally mis-phrased the revision to give this impression. The motivation for the current analyses and the article was rather to take up the opportunity provided by new deep-learning systems that transform sounds to words with good accuracy to see whether they can model neural activity that is outside the bounds of prior computational models of speech processing – and in particular address components of the speech recognition process that context invariant categorical models cannot in themselves address.

We have revised segments of the Introduction and Discussion in attempt to clarify this and more explicitly define phonemes. We acknowledge in the Discussion that it isn’t straightforward to peg components of deep-learning systems to components of theoretical models such as Hickok and Poeppel (2007) and reference Tezcan et al. and Mai et al. in the Intro alongside Di Liberto et al. in using combining phoneme and spectrogram data to predict neurophysiological responses (Thanks for those refs).

We have pasted the new text (red font this revision, green last revision, black original text).

**Introduction**

The apparent ease with which the human brain transforms speech sounds into words belies the complexity of the task. This complexity is due in large part to speech variability - each time a word is spoken, the sound is different. Speech variability is most striking in extreme cases such as when people have unfamiliar accents, shout, whisper or sing, but is always present to some degree, even when the same person repeats the same phrase (Liberman et al. 1967, Smith et al. 1995). How brains transform such variable speech sounds into language is a key unresolved question in cognitive neuroscience. By enabling temporally precise estimates of brain activity, electrophysiological measures such as scalp EEG have provided evidence that the brain transforms natural continuous speech into words as a cascading process with abstract categorical speech units such as phonemes or their articulatory features serving as intermediary pre-lexical representations (di Liberto et al. 2015, Gilles et al. 2023, Tezcan et al. 2023, Mai et al. 2024). The transformation to phoneme sequences prospectively enables the brain to distinguish word identity, independent of the speaker, intonation, volume, word meaning and so on. To support this, researchers have typically revealed how EEG models of speech comprehension are improved when representing the speech stimulus as a time-series of categorical phoneme feature vectors in addition to the audio signal (e.g. di Liberto et al. 2015). However, as observed by Daube et al. (2019): (1) The phoneme predictive advantage revealed by di Liberto et al. (2015) can more parsimoniously be explained by phoneme timing rather than phoneme identity or articulatory structure, and phoneme timing can be approximated by estimating the derivative of speech energy. (2) As a broader issue, it is desirable to produce more complete models of the speech recognition process, because categorical models typically do not specify the computational mechanism that categorizes variable speech sounds, nor how sub-word categories are derived from audio data. They therefore may overlook key components of the speech recognition process in the brain.

**Discussion**

The current study has revealed electrophysiological correlates of the linguistic transformation of heard speech using the end-to-end speech recognition model Whisper. This addresses a gap in previous work that has typically relied upon hand-crafted context invariant categorical speech units such as phonemes to capture an intermediary phase between sound and words, without modeling the mechanism that maps sounds to categories, and potentially also the representations invoked in this mapping. The current results suggest that (1) EEG is better predicted with the more complete model provided by Whisper. (2) Whisper helps reveal neural correlates of a contextualized speech transformation that reflects both prelexical and lexical representation and predictive processing which cannot be modeled by context invariant categorical approaches (by definition). To strengthen the case that the newly predicted EEG signal reflects a linguistic transformation as opposed to a brain-like filter of concurrent acoustic speech, the study further demonstrated that Whisper correlates were sensitive to listener attention. Specifically, correlates of Whisper’s deeper more linguistic layers selectively diminished comparative to early layers when listeners ignored one speaker in favor of listening to another (for whom the correlates of deep layers were present). More generally, this study exemplifies how deep-learning models can help tackle unresolved questions in human speech comprehension, and in so doing predict neurophysiological data with minimal experimenter intervention.

The flipside of the EEG modelling benefits observed above is that interpreting deep-learning models and what they predict in brain data are both notoriously challenging, because internal representations require empirical investigation to interpret and may neatly not align with spectrotemporal, phonological, lexical and combinatorial components of theoretical models (e.g. Hickok and Poeppel, 2007). Nonetheless, the inputs, outputs, architecture and training objective(s) of a deep learning model provide initial clues to how information is coded and reshaped throughout the network, and therefore what may drive EEG prediction. To recap Whisper is trained to translate multilingual speech spectra into words in either the input language or English and to also annotate word timing (see also **Methods**). The network architecture deploys a Transformer Encoder to contextualize and transform speech spectrograms into time-aligned vector representations (these vectors were the basis of the current study) and then a Transformer Decoder to transcribe the Encoder output vectors (which we did not examine here, please see **Methods** and **Introduction**). Thus, our starting assumption was that Whisper’s Encoder gradually transforms speech into a contextualized linguistic representation – which may be language invariant (perhaps even encoding semantics) because the Encoder output is optimized to prepare speech for transcription either within or between languages. However, at this stage the precise nature of network representations still remained ambiguous.

Also, later in the Discussion

In light of the above, we examined the impact of limiting Whisper’s opportunity to form predictions, by limiting speech context. Despite our anticipation that EEG prediction accuracy might monotonically improve or asymptote with longer contexts (up to the 30s max), we found 5-10s context to be most accurate (~20% extra variance predicted than .5s). Critically the 5-10s contextual advantage provides evidence that EEG encodes information across multiword speech contexts, and that contextualized models predict components of EEG that context invariant categorical models cannot capture. ~~Critically the 5-10s contextual advantage provides evidence that EEG encodes information across multiword speech contexts, and thereby questions the completeness of categorical speech models that are context invariant.~~ However, it remains unclear why 5-10s speech is advantageous and future work will be needed to characterize EEG correlates of speech contextualization at lexical and prelexical levels. One approach could be examining how representations in speech models vary in the presence/absence of linguistic contexts that do/don’t make upcoming words and sounds predictable (e.g. Pouw et al. 2024) and then testing whether model differences capture corresponding differences in electrophysiological responses to the predictable/unpredictable stimuli.

Given the availability of different speech models, one might wonder whether Whisper is currently the “best” model of brain activity. We consider that the answer to this question must in part be borne out experimentally through correlations between models and brain data. However, besides this, a study’s aims may determine the appropriate model for use. For instance, Whisper’s access to a preconfigured cross-lingual text vocabulary renders it an unsuitable model of childhood language acquisition when the word vocabulary also must be learnt (see also Millet et al. 2022). We chose to focus on Whisper because it provides an accurate end-to-end transformation of audio to text and may therefore discover intermediary pre-lexical and/or lexical representations from audio data that could help predict brain responses. ~~We chose to focus on Whisper because it provides an accurate end-to-end transformation of audio to text, and the potential to discover intermediary pre-lexical representations from audio data that have high utility for speech recognition. Thus, we considered it a good vehicle to address limitations of traditional hand-crafted and context-invariant categorical approaches that do not model how audio is categorized (Daube et al. 2019). Even though Whisper is trained with access to word units, we considered these to be more ecologically relevant units than phonemes - which humans don’t explicitly need to know about to communicate.~~

**2. Replicate DiLiberto et al. (2015)**

Thank you for adding a figure on the performance of categorical features relative to Whisper features to the text. I do think it’s a bit odd that it’s in the Supplementary Materials though. I

understand you weren’t convinced it was necessary, but since the abstract, introduction, and discussion of the revised paper all continue to focus on the advantages of Whisper over categorical models, it seems a bit strange that the only figure addressing categorical models is in the Supplementary Materials.

That choice aside, it’s not clear to me from Supplementary Figure 7 whether you were able to replicate DiLiberto et al.’s (2015) results. This for me is really important, especially considering the inability of the 80-feature spectrographic model to outperform the Env&Dv model. It got me thinking more generally about differences between your pipeline and DiLiberto et al.’s, and I realized that your choice of sampling rate may be adversely affecting the performance of your more time-sensitive feature sets (i.e., spectrographic features and phones).

In particular, it looks like work in this area that uses higher sample rates (>100Hz) shows more sensitivity to these feature types generally (e.g., DiLiberto et al. 2015, Gwilliams et al. 2022, Tezcan et al. 2023, Mai et al. 2024), compared to those using lower sample rates (e.g., this manuscript, Daube et al. 2019). This shouldn’t be surprising considering that some phonemes are as little as 10ms in duration, and many of the acoustic correlates of phoneme identity (e.g., formant transitions) are similarly brief. With a sampling resolution of 32Hz, your time bins are over 30ms, which means that they don’t include information that we know is important for speech processing.

What I would say is: try to replicate the content of DiLiberto et al.’s Figure 2A,B with your signal and features at 32Hz and 128Hz. If the results are qualitatively similar, then report that in the Supplementary Materials, and put somewhere in the text that you’ve validated that your pipeline parameters are reasonable for the features and data you’re using. However, if DiLiberto et al.’s results can’t be qualitatively validated at 32Hz, then you’ll need to redo all analyses using an appropriate sampling rate.

Regardless of the outcome of the replication, there should be text in the discussion section addressing how the results of this work compare/contrast to DiLiberto et al.’s and the implications of that.

Thanks for this.

We hope that that the revisions made in response to the last point clarify that our goal was never to produce a “phoneme bashing” paper, and a major reason for excluding the phoneme model from analyses was that Daube et al (2019) had shown on the same data that acoustic measures (which we did model) more parsimoniously account for the phoneme advantage. On these grounds we did not and do not emphasize the phoneme results in the main article and we continue not to compare and contrast our results to the phoneme results in the Discussion. This is because it may be laboring on a moot point given that it is contentious whether this current EEG dataset reflects phonemes, irrespective of whether they are encoded in the brain.

Nonetheless, we have repeated Supplementary Figure 7 with the EEG data sampled at 128Hz, the phoneme articulation model at 128Hz and with Whisper layers now up-sampled from 32Hz to 128Hz. The outcome is much the same, albeit that the phoneme model does have a predictive advantage over Whisper L0, which it didn’t have in the original 32Hz analysis. It is possible that the up-sampling disadvantages Whisper here, but the key finding that Whisper accounts for the phoneme features is the same.

To give further confidence that the current pipeline can replicate Di Liberto’s initial results – which used a phoneme model and a 16 channel spectrogram – we repeated the 2015 analysis with the current pipeline. Rather than attempting to directly replicate Figure 2 (which used 10 rather than the current 19 participants and analysed selected ROIs), we deployed the variance partitioning approach used throughout this battery of analyses. This replication is not included in the article but is pasted into the response below Supp Figure 7.

Supplementary Figure 7 is referenced in the manuscript as:

For completeness, **Supplementary Figure 7** presents a supporting analysis showing that Whisper Layers 1-6 model EEG features predicted by a phoneme articulation model (Di Liberto et al. 2015), though these same EEG features may reflect acoustic measures (Daube et al. 2019).

**Supplementary Figure 7 (Figure 2 Companion).** Whisper captures almost all information that is useful for predicting the current EEG data in a phoneme articulation model. The phoneme articulation model contains 19 binary articulatory features used in Di Liberto et al. (2015). EEG data was resampled at 128Hz to match the articulatory modeling set up of Di Liberto et al. (2015), and the 32Hz Whisper models used elsewhere in this article were up sampled to 128Hz. Time-lags used in fitting temporal response functions to both the phoneme articulation model and Whisper were 0-250ms to match the 2015 analysis. With this set up, predicted variance partitioning analyses found that Whisper layers 1 to 6 could each account for the EEG predictions made by the articulation model.

The phoneme articulation vs 16 channel spectrogram results replicating di Liberto et al. (2015) are illustrated below.

**16 Channel Spectrogram**	**Phoneme Articulation**

Thanks for your careful attention to this review!
